# A CD44/Brg1 nuclear complex confers mesenchymal progenitor cells with enhanced fibrogenicity in idiopathic pulmonary fibrosis

**DOI:** 10.1172/jci.insight.144652

**Published:** 2021-05-10

**Authors:** Libang Yang, Hong Xia, Karen Smith, Adam Gilbertsen, Daniel Beisang, Jonathan Kuo, Peter B. Bitterman, Craig A. Henke

**Affiliations:** 1 Department of Medicine and; 2 Department of Pediatrics, University of Minnesota, Minneapolis, Minnesota, USA.

**Keywords:** Pulmonology, Fibrosis

## Abstract

Idiopathic pulmonary fibrosis (IPF) is a progressive fibrotic lung disease. We previously identified fibrogenic mesenchymal progenitor cells (MPCs) in the lungs of patients with IPF who serve as drivers of progressive fibrosis. Recent single-cell RNA sequencing work revealed that IPF MPCs with the highest transcriptomic network entropy differ the most from control MPCs and that increased CD44 was a marker of these IPF MPCs. We hypothesize that IPF MPCs with high CD44 (CD44^hi^) expression will display enhanced fibrogenicity. We demonstrate that CD44-expressing MPCs are present at the periphery of the IPF fibroblastic focus, placing them in regions of active fibrogenesis. In a humanized mouse xenograft model, CD44^hi^ IPF MPCs are more fibrogenic than CD44^lo^ IPF MPCs, and knockdown of CD44 diminishes their fibrogenicity. CD44^hi^ IPF MPCs display increased expression of pluripotency markers and enhanced self-renewal compared with CD44^lo^ IPF MPCs, properties potentiated by IL-8. The mechanism involves the accumulation of CD44 within the nucleus, where it associates with the chromatin modulator protein Brahma-related gene 1 (Brg1) and the zinc finger E-box binding homeobox 1 (Zeb1) transcription factor. This CD44/Brg1/Zeb1 nuclear protein complex targets the Sox2 gene, promoting its upregulation and self-renewal. Our data implicate CD44 interaction with the epigenetic modulator protein Brg1 in conveying IPF MPCs with cell-autonomous fibrogenicity.

## Introduction

Idiopathic pulmonary fibrosis (IPF) is the most prevalent and deadly interstitial lung disease, causing up to 40,000 deaths each year in the United States (1, 2). Despite major advances in our understanding of disease initiation centered around pathological changes in the alveolar epithelium, IPF remains a major unsolved clinical problem with a median survival of 3 to 5 years (3). One reason for this is that currently available anti-fibrotic agents slow, but do not arrest, fibrotic progression (3). To arrest fibrotic progression, its obligatory drivers need to be identified. Recently, we made several discoveries studying primary cells and extracellular matrix (ECM) from patients with IPF that provide a path forward toward understanding mechanisms that drive fibrosis following disease initiation (4–9). In addition to canonical disease-drivers downstream of alveolar epithelial injury, fibrosis progression in IPF involves both cell-autonomous and ECM-driven mechanisms. As in cancer, in IPF there is cooperation between autonomous cells and their microenvironment.

Cell-autonomous fibrogenicity was established when we discovered intrinsically fibrogenic MPCs in the lungs of patients with IPF that are one source of IPF fibroblasts (4, 5, 9). IPF MPCs display a distinct transcriptome, cause nonresolving interstitial lung fibrosis in a humanized mouse xenograft model and are found concentrated in a highly cellular region on the perimeter of the fibroblastic focus in IPF lung tissue. IPF MPCs possess high levels of S100A4 that support their self-renewal and are required for their fibrogenicity. However, the mechanism of cell-autonomous fibrogenicity remains unclear. To gain insight, we performed single-cell RNA-Seq of IPF and control MPCs. Analysis of the genome-wide transcriptome revealed that the cells segregated on the basis of disease tag, confirming our bulk RNA-Seq studies demonstrating that IPF MPCs have a transcriptome that is distinct from control MPCs (10). As an unbiased approach to stratifying the cells, we chose transcriptional network entropy, which serves as a quantitative measure of a cell’s differentiated state, with higher entropy indicating a less differentiated state (11, 12). Our data revealed heterogeneity among IPF MPCs without discrete subpopulations (10). The most highly entropic IPF MPCs displayed the greatest differences in their transcriptional profile compared with control MPCs. These results indicate that IPF MPCs acquire a pathological phenotype early in their differentiation and suggest that this distinct transcriptome may underlie their fibrogenic phenotype. When examining our data for genes that best distinguished the most highly entropic IPF MPCs, we identified CD44^hi^ expression (10). Together, these data raise the possibility that IPF MPCs with the highest expression of CD44 may display the most robust fibrogenic phenotype.

To test this, we used FACS to physically separate IPF MPCs with CD44^hi^ expression from those with low CD44 expression (CD44^lo^). Here we show that CD44-expressing MPCs are present at the periphery of IPF fibroblastic foci in human IPF lung tissue, indicating that they are located in regions with active fibrogenesis. Utilizing our humanized mouse xenograft model (5), we demonstrate that CD44^hi^ IPF MPCs display more robust fibrogenicity in vivo compared with CD44^lo^ IPF MPCs. Furthermore, CD44^hi^ IPF MPCs display increased expression of pluripotency markers and greater self-renewal in vitro. Knockdown of CD44 in CD44^hi^ IPF MPCs reduces their in vitro self-renewal capacity and diminishes their fibrogenicity in vivo. CD44^hi^ IPF MPCs display increased expression of the IL-8 receptor CXCR1 and increased expression and secretion of IL-8 compared with CD44^lo^ IPF MPCs. Exogenous IL-8 markedly increases the expression of Sox2 and potently stimulates CD44^hi^ IPF MPC self-renewal. The mechanism involves an IL-8–mediated increase in CD44 expression, nuclear accumulation of full-length CD44 and the association of CD44 with the chromatin remodeler protein Brahma-related gene 1 (Brg1) (also termed SMARCA4) to form a nuclear protein complex that interacts with the Zeb1 transcription factor to induce Sox2 gene expression. Taken together, these data indicate that a CD44 interaction with the epigenetic modulator protein Brg1 conveys IPF MPCs with cell-autonomous fibrogenicity.

## Results

### CD44-expressing IPF MPCs are present at the periphery of the fibroblastic focus.

**Figure 1:**
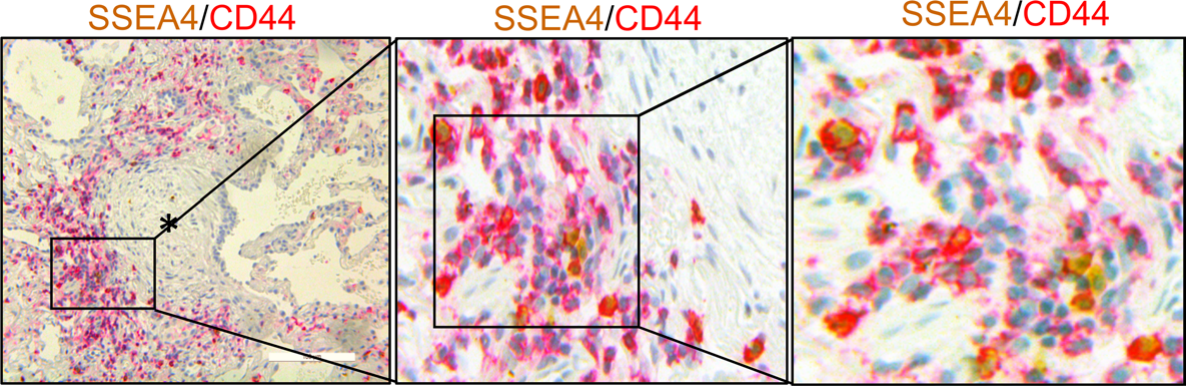
CD44-expressing IPF MPCs are present at the periphery of the fibroblastic focus. IHC double staining showing SSEA4 positive (brownish-yellow) MPCs in the active fibrotic front expressing CD44 (red). Asterisk denotes focus (myofibroblast) core. Scale bar: 100 μm.

Our single cell RNA sequencing (scRNA-Seq) data indicate that CD44 is a marker of highly entropic IPF MPCs (10). Based on this information, we first performed IHC double staining of human IPF lung tissue to examine the distribution of CD44-expressing MPCs (double positive for SSEA4 and CD44). CD44-expressing MPCs were found concentrated at the periphery of the fibroblastic focus, with only a few CD44-expressing cells in the focus (myofibroblast) core (Figure 1 and Supplemental Figure 1; supplemental material available online with this article; https://doi.org/10.1172/jci.insight.144652DS1). These findings are consistent with our previous published results demonstrating SSEA4 + MPCs concentrated on the perimeter of the fibroblastic focus in human IPF lung tissue and are in contrast to our findings that in control lung tissue, SSEA4-expressing cells, while being observed in small and large airways and vascular structures, are absent in alveolar walls (4, 5). These data place CD44-expressing MPCs in regions previously documented to be loci of fibrogenesis (5–8), consistent with a role for these cells in fibrotic progression.

### CD44^hi^ IPF MPCs are more fibrogenic than CD44^lo^ IPF MPCs.

**Figure 2:**
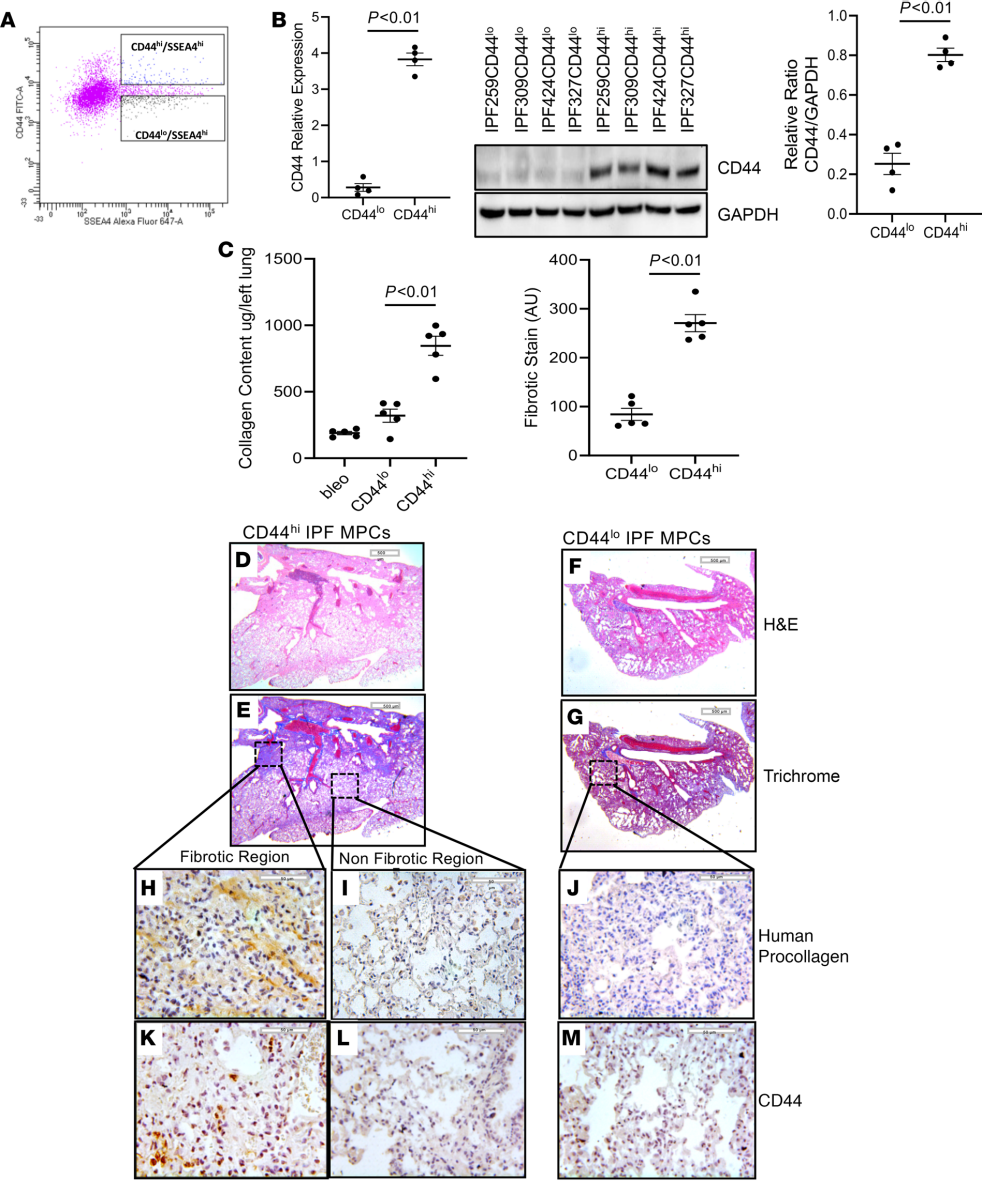
CD44^hi^ IPF MPCs are more fibrogenic than CD44^lo^ IPF MPCs. (**A**–**E**) NSG mice were treated with IT bleomycin. Two weeks later, mice received either CD44^hi^ or CD44^lo^ IPF MPCs (IPF424); 10 mice/group. Flow cytometry showing isolation of SSEA4^hi^/CD44^hi^ and SSEA4^hi^/CD44^lo^ IPF MPCs (**A**). CD44 levels were quantified in CD44^hi^ and CD44^lo^ IPF MPCs by qPCR (**B**; left panel) and Western blot analysis (**B**; middle panel) (*n* = 4 cell lines tested IPF259, IPF309, IPF424, and IPF327); densitometry values summarize Western blot data (right panel). Lungs were harvested at week 6. (**C**) Collagen content was quantified in left lungs by Sircol assay (left panel). Ten mice from the above animal experiment (5 mice each from CD44^hi^ and CD44^lo^ groups; IPF 424) were used in this analysis. Semiquantitative analysis of collagen deposition was performed by Trichrome staining (right panel). Three sections from each animal were screened. Three Trichrome stained images at low power (5×; scale bar: 500 µm) randomly selected from each section were used for quantification. Blue regions (fibrotic stain) were defined and quantified using ImageJ (right panel). (**D**–**M**) Serial 4 µm sections of right lung tissue from mice receiving CD44^hi^ IPF MPCs (**D**–**G**; scale bar: 200 µm) (**H**–**M**; scale bar: 50 µm). H&E and Trichrome stains assessing fibrosis and collagen deposition, respectively (**D**–**G**). IHC using an antibody recognizing human procollagen to identify human cells and assess collagen synthesis (**H**–**J**) and a CD44 antibody to determine the distribution of CD44 expressing cells (**K**–**M**). (**H**, **I**, **K**, and **L**) IHC for procollagen (**H** and **I**) and CD44 (**K** and **L**) to assess the distribution of human cells expressing collagen and CD44-expressing cells from fibrotic and nonfibrotic lung regions from mice receiving CD44^hi^ IPF MPCs. Data expressed as mean ± SEM. *P* values were determined by 2-tailed Student’s *t* test.

Our scRNA-Seq data indicate that IPF MPCs with the highest transcriptional network entropy differ the most from control MPCs (10). Since CD44 is a marker of the most highly entropic IPF MPCs, we reasoned that those IPF MPCs with the highest expression of CD44 may be more fibrogenic than IPF MPCs with lower CD44 expression. To test this, we utilized our humanized mouse xenograft model (5). In this system, administration of human IPF MPCs to NSG mice pretreated with intratracheal bleomycin results in nonresolving interstitial lung fibrosis. CD44^hi^ or CD44^lo^ IPF MPCs were isolated by flow cytometry selecting for SSEA4^hi^/CD44^hi^ and SSEA4^hi^/CD44^lo^ cells (Figure 2A). Verification of elevated CD44 levels in CD44^hi^ IPF MPCs was performed by quantitative PCR (qPCR) and Western blot analysis (Figure 2B). CD44 levels in CD44^hi^ IPF MPCs were elevated 13-fold by qPCR and increased 4.5-fold by Western blot analysis compared with CD44^lo^ IPF MPCs. To initiate an experiment, NSG mice were first treated with low-dose intratracheal bleomycin. Two weeks later they received CD44^hi^ or CD44^lo^ MPCs via the tail vein. Lungs were harvested 4 weeks later. Collagen content was 55% higher in the lungs of mice receiving CD44^hi^ IPF MPCs compared with mice receiving CD44^lo^ IPF MPCs (871 ± 83 μg/left lung versus 450 ± 61 μg/left lung) (Figure 2C). H & E and trichrome staining demonstrated more extensive fibrosis with corresponding collagen deposition in the lungs of mice receiving CD44^hi^ IPF MPCs (Figure 2, D and E) compared with mice receiving CD44^lo^ MPCs (Figure 2, F and G). Of note, we analyzed the engraftment of human CD44^hi^ or CD44^lo^ IPF MPCs in the lungs of the immune-deficient mice using a real-time PCR method shown to be sensitive for the detection and quantification of human cells in mice (13). Using this method, we found no significant difference in the engraftment of CD44^hi^ or CD44^lo^ IPF MPCs (Supplemental Figure 2). This indicates that both groups received equal numbers of cells and that the difference in fibrosis between the groups resulted from different biological properties of CD44^hi^ and CD44^lo^ cells. In the lungs of mice receiving CD44^hi^ IPF MPCs, regions with increased collagen deposition contained numerous human cells expressing procollagen (Figure 2H and Supplemental Figure 3). References to location of panels are to be avoided in main text as figure layout is subject to change. “Upper left panel” here and all subsequent references to panel location have been deleted.), whereas anatomically uninvolved regions were devoid of collagen deposition and human cells expressing procollagen (Figure 2I), implicating the human CD44^hi^ IPF MPCs with fibrosis development. The lungs of mice receiving CD44^lo^ IPF MPCs displayed few human cells expressing procollagen (Figure 2J and Supplemental Figure 3). Interestingly, while numerous human cells expressing procollagen were present in the fibrotic regions in the lungs of mice receiving CD44^hi^ IPF MPCs, only a few CD44 staining cells were seen in these areas (Figure 2K). A relative paucity of CD44-expressing cells were present in nonfibrotic regions in the lungs of mice that received CD44^hi^ IPF MPCs as well in the lungs of mice that were administered CD44^lo^ IPF MPCs (Figure 2, L and M). We previously found that IPF MPCs lose the progenitor marker SSEA4 as they differentiate to IPF fibroblasts over a 4 week time period in vitro (4). Similarly, we found that CD44 expression in CD44^hi^ IPF MPCs also declined as a function of time in vitro (Supplemental Figure 4). Taken together, these data support the concept that during their 4 week residence in the mouse lung, human CD44^hi^ IPF MPCs lose CD44 expression as they differentiate into procollagen-expressing fibroblasts. This interpretation is consistent with the IHC staining pattern seen in human IPF lung tissue, where SSEA4+/CD44+ MPCs are present at the periphery of the fibroblastic focus; whereas few cells expressing SSEA4 or CD44 are present in the focus core containing procollagen-expressing myofibroblasts (see Figure 1) (7, 8, 14).

### CD44 regulates the fibrogenicity of CD44^hi^ IPF MPCs.

**Figure 3:**
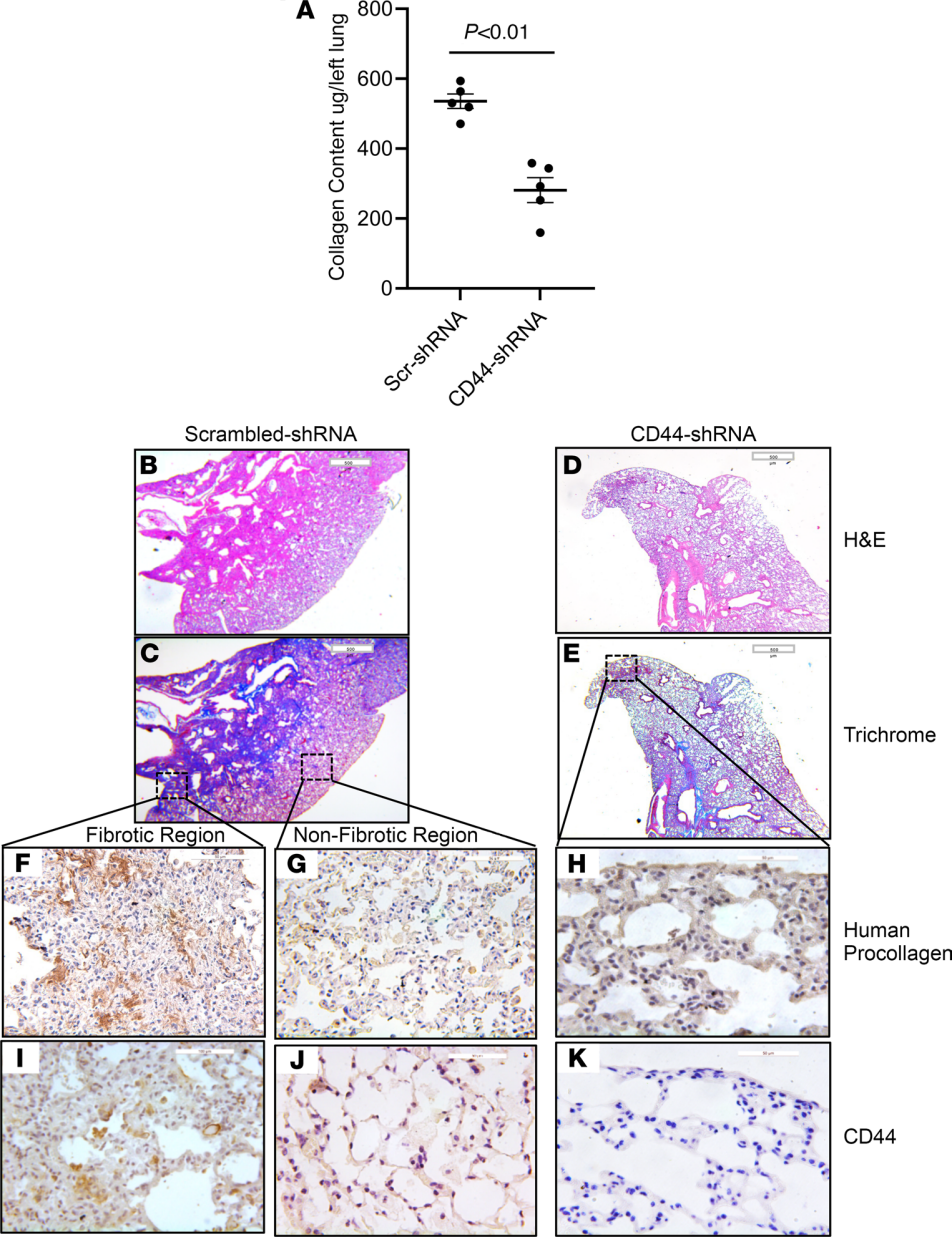
CD44 regulates the fibrogenicity of CD44^hi^ IPF MPCs. NSG mice were treated with IT bleomycin (1.25 U/kg). Two weeks later, the mice received IPF MPCs (IPF424) transduced with either CD44 shRNA or scrambled shRNA via tail vein injection (10^6^ cells/100 μL); 10 mice/group. Lungs were harvested 4 weeks after cell administration. (**A**) Collagen content was quantified in left lungs by Sircol assay. (**B**–**K**) Serial 4 μm sections of right lung tissue from mice receiving CD44^hi^ IPF MPCs transduced with scrambled-shRNA or CD44-shRNA (**B**–**E** scale bar: 500 μm; **F**, **G**, **I**, and **J** scale bar: 50 μm; **H** and **K** scale bar: 20 μm). Representative H&E and Trichrome stains assessing fibrosis and collagen deposition, respectively (**B**–**E**). IHC using an antibody-recognizing human procollagen to identify human cells and assess collagen synthesis (**F**–**H**) and a CD44 antibody to determine the distribution of CD44-expressing cells (**I**–**K**). (**F**, **G**, **I**, and **J**) IHC for human procollagen (**F** and **G**) and CD44 (**I** and **J**) to assess the distribution of human cells expressing collagen and CD44-expressing cells from fibrotic and nonfibrotic lung regions from mice receiving CD44^hi^ IPF MPCs transduced with scrambled shRNA. Data are expressed as mean ± SEM. *P* values in **A** were determined by 2-tailed *t* test.

Although our data indicate that CD44^hi^ IPF MPCs are more fibrogenic than CD44^lo^ IPF MPCs, it remained unclear whether CD44 is only a marker of fibrogenic IPF MPCs or whether CD44 itself plays a mechanistic role in their fibrogenicity. To examine whether CD44 regulates CD44^hi^ IPF MPC fibrogenicity, we conducted knockdown experiments. CD44^hi^ IPF MPCs were transduced with either CD44 shRNA or scrambled shRNA. CD44 shRNA decreased CD44 expression 82% compared with scrambled shRNA (Supplemental Figure 5). Transduced cells were tested for fibrogenicity in the humanized mouse model. Knockdown of CD44 reduced CD44^hi^ IPF MPC fibrogenicity by 39% (collagen content 326 ± 48 μg/left lung versus 533 ± 32 μg/left lung) (Figure 3A). Consistent with this, large fibrotic regions were present in the lungs of mice receiving CD44^hi^ IPF MPCs transduced with scrambled shRNA (Figure 3, B and C). In contrast, sparse regions of fibrosis with accompanying collagen deposition were present in the lungs of mice receiving MPCs in which CD44 had been knocked down (Figure 3, D and E). IHC analysis of lung tissue from mice receiving CD44^hi^ IPF MPCs transduced with scrambled shRNA contained numerous human cells expressing procollagen in fibrotic regions (Figure 3F), whereas anatomically uninvolved regions were devoid of human cells expressing procollagen (Figure 3G). In contrast, IHC analysis demonstrated few human cells expressing procollagen I in the lungs of mice receiving MPCs in which CD44 had been knocked down (Figure 3H). While some CD44-expressing cells were present in the fibrotic regions of control mice (Figure 3I), very few CD44-expressing cells were found in nonfibrotic regions of control mice (Figure 3J) or in the lungs of CD44 knockdown mice (Figure 3K). Together, these data indicate that CD44^hi^ IPF MPCs display a robust fibrogenic phenotype and that CD44 is involved in the mechanism.

### CD44^hi^ IPF MPCs display increased expression of pluripotency markers and greater self-renewal capacity.

**Figure 4:**
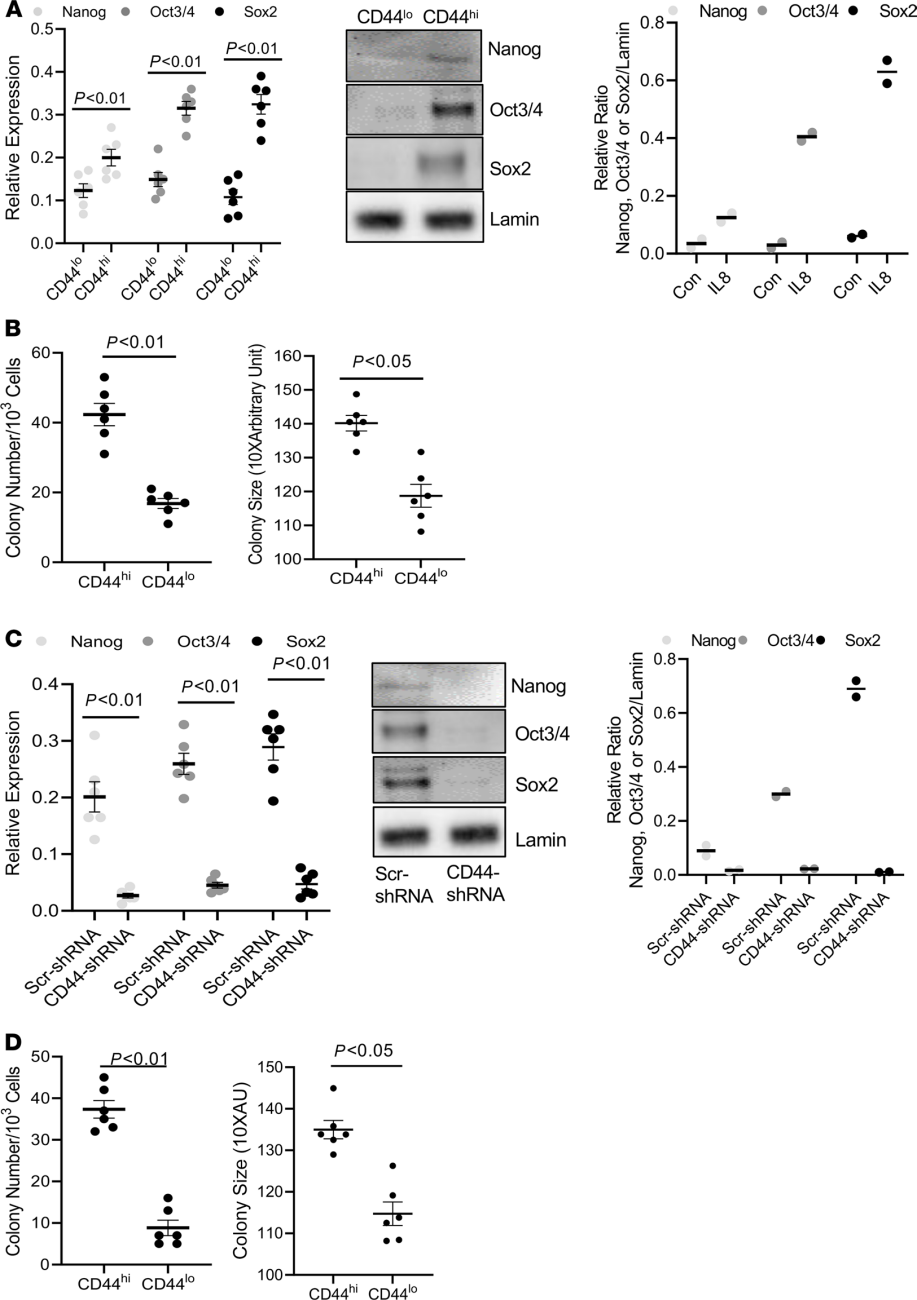
CD44^hi^ IPF MPCs display increased expression of pluripotency markers and greater self-renewal capacity. (**A**) Oct3/4, Nanog, and Sox2 expression was quantified in nuclear fractions of CD44^hi^ ^and^ ^lo^ IPF MPCs by qPCR (left panel) and Western Blot analysis (middle panel). Densitometry values summarizing Western blot data in right panel **B**. Self-renewal was quantified in an anchorage-independent colony-forming assay. (**C** and **D**) CD44 was knocked down in CD44^hi^ IPF MPCs using CD44 shRNA (CD44-shRNA). Scrambled shRNA (Scr-shRNA) served as control. Oct3/4, Nanog, and Sox2 expression were quantified by qPCR (left panel) and Western Blot analysis (middle panel). Densitometry values summarizing Western blot data in right panel **C**. Self-renewal was assessed in the colony-forming assay (**D**). For **C** and **D**, IPF 422 and IPF424 were used. All data are shown as mean ± SEM; *n* ≥ 3 independent experiments for each condition or group except the Western blot is from a single experiment representative of 2 independently conducted replicates. Data are expressed as mean ± SEM. *P* values were determined by 2-tailed Student’s *t* test.

Since CD44 is a marker of highly entropic IPF MPCs and high entropy indicates a less differentiated state, we next examined CD44^hi^ and CD44^lo^ IPF MPCs for expression of pluripotency markers and their ability to self-renew in a colony-forming assay. Consistent with their high entropy, CD44^hi^ IPF MPCs displayed higher protein levels of pluripotency transcription factors Oct3/4 (23% higher), Nanog (13% higher), and Sox2 (61% higher) (Figure 4A) as well as greater self-renewal capacity (Figure 4B) compared with CD44^lo^ IPF MPCs. Knockdown of CD44 reduced the expression of Oct3/4, Nanog, and Sox2 (Figure 4C) and decreased colony formation (Figure 4D), indicating that CD44 plays a role in regulating the progenitor status of CD44^hi^ IPF MPCs and their ability to self-renew.

### The CXCR1/IL-8 axis regulates CD44^hi^ IPF MPC self-renewal.

**Figure 5:**
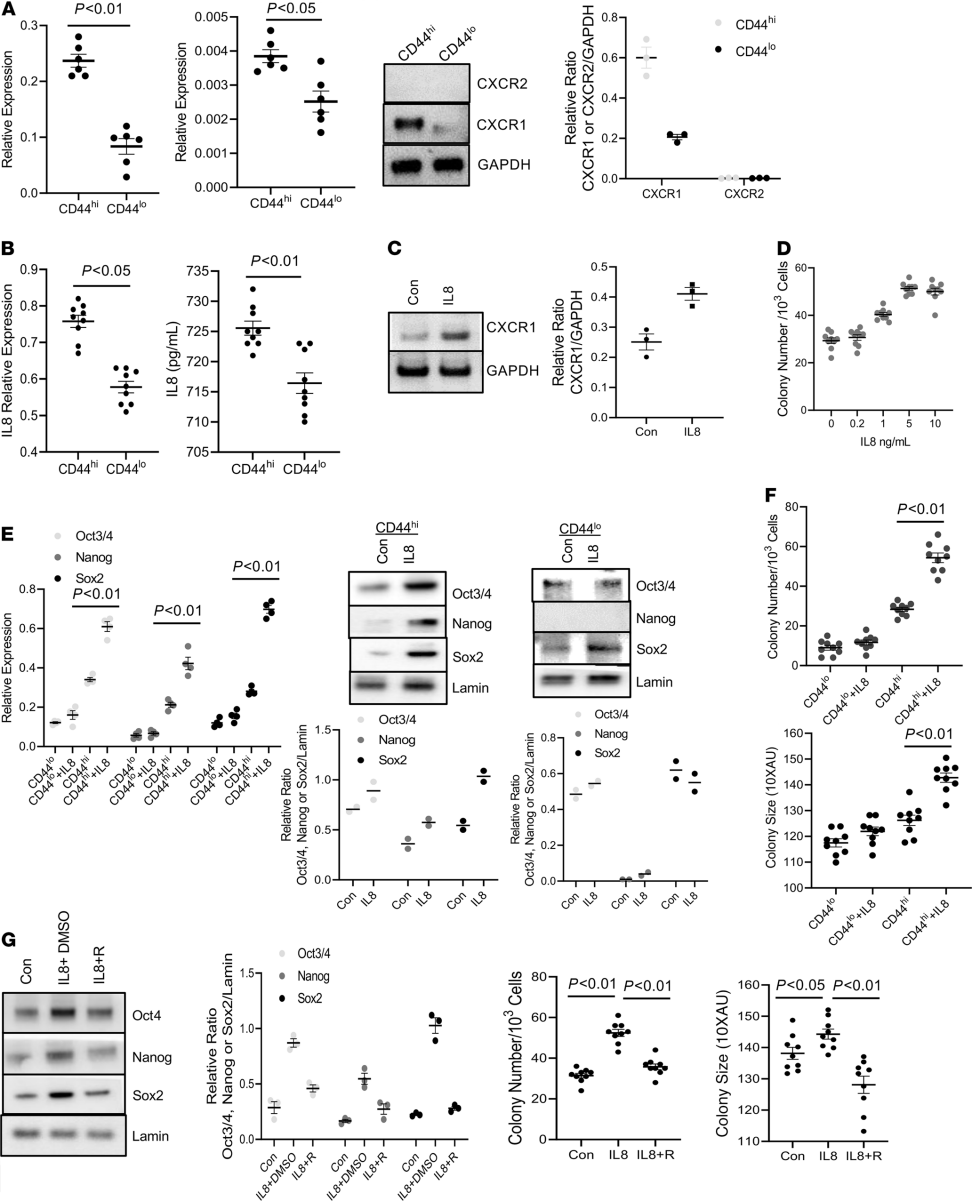
The CXCR1/IL-8 axis regulates CD44hi IPF MPC self-renewal. (**A**) CXCR1/2 expression was quantified in CD44^hi^ and CD44^lo^ IPF MPCs by qPCR (left panels) and Western blot analysis (right middle panel; densitometry values right panel). *n* = 2 cell lines tested (IPF 422 and 424). (**B**) IL-8 expression was quantified by qPCR (left panel). IL-8 secretion was quantified by ELISA (right panel). *n* =3 cell lines tested (IPF 422, 424, and 442). (**C**) CD44^hi^ IPF MPCs (IPF 422, 424, and 442) were treated with recombinant IL-8 or control (Con). CXCR1 expression was quantified by Western blot analysis (densitometry values right panel). (**D**) CD44^hi^ IPF MPCs (IPF 422, 424, and 442) incorporated into methylcellulose gels were treated with IL-8. Colony number quantified. (**E**) CD44^hi^ and CD44^lo^ IPF MPCs (IPF 422 and 424) were treated with IL-8. Oct3/4, Nanog, and Sox2 expression was quantified by qPCR (left panel) and Western blot analysis (right 2 panels; densitometry values below Western blots). (**F**) CD44^hi^ and CD44^lo^ IPF MPCs (IPF 422, 424, and 442) incorporated into methylcellulose gels were treated with IL-8. Shown is colony-forming assay. (**G**) CD44^hi^ IPF MPCs (IPF 422, 424, and 442) were treated with IL-8 in the presence of the IL-8 receptor inhibitor Reparixin (IL-8 + R) or vehicle (IL-8 + DMSO). Cells not treated with IL-8 served as a control (Con). Sox2, Nanog, and Oct3/4 levels were quantified by Western blot analysis (left panels, densitometry values left middle panel). Colony-forming assay (right panels). *n* ≥ 3 independent experiments for each experimental condition except **E**, where *n* = 2 independent experiments were performed, and Western blot, which is from a single experiment representative of 3 independently conducted replicates. Data are expressed as mean ± SEM. *P* values determined by 2-tailed Student’s *t* test, except in **G**, where **P** values were determined by 1-way ANOVA.

We previously reported that compared with control MPCs, IPF MPCs display increased steady state levels of IL-8 and its cognate receptor CXCR1 and secrete more IL-8 (9). IL-8 functions in an autocrine manner promoting IPF MPC self-renewal. Since CD44^hi^ IPF MPCs display greater self-renewal capacity compared with CD44^lo^ IPF MPCs, we next examined CD44^hi^ and CD44^lo^ IPF MPCs for CXCR1 and CXCR2 expression as well as IL-8 expression and secretion. CD44^hi^ IPF MPCs displayed an approximately 3-fold–increased protein expression of CXCR1 compared with CD44^lo^ IPF MPCs (Figure 5A). CXCR2 protein levels were expressed below the limit of detection in both CD44^hi^ and CD44^lo^ IPF MPCs. CD44^hi^ IPF MPCs expressed and released more IL-8 compared with CD44^lo^ IPF MPCs (Figure 5B; left and right panels, respectively). Furthermore, CXCR1 expression was modestly increased (38%) in CD44^hi^ IPF MPCs treated with IL-8 (Figure 5C). Together, these data suggest that the highly entropic CD44^hi^ IPF MPCs with increased CXCR1 levels are primed to respond to IL-8 in the fibrogenic niche at the periphery of the fibroblastic focus where they self-renew.

IL-8 promotes cancer stem cell self-renewal, and we found that IL-8 also stimulates IPF MPC self-renewal (9, 15). Since CD44^hi^ IPF MPCs express and secrete greater amounts of IL-8 and express higher levels of CXCR1 compared with CD44^lo^ IPF MPCs, we next examined the effect of exogenous IL-8 on the self-renewal of CD44^hi^ ^and^ ^lo^ IPF MPCs. Exogenous IL-8 increased the self-renewal of CD44^hi^ IPF MPCs in a dose-dependent fashion, with a maximum effect at 5 ng/mL of IL-8 (Figure 5D). We next compared the effect of IL-8 on pluripotency marker expression and the self-renewal of CD44^hi^ ^and^ ^lo^ IPF MPCs. IL-8 increased the protein expression of Sox2 (56% increase), Nanog (45% increase), and Oct3/4 (15% increase) in CD44^hi^ IPF MPCs with the greatest effect on Sox2 expression. IL-8 had no appreciable effect on Nanog expression and modest effects on Sox2 and Oct3/4 expression in CD44^lo^ IPF MPCs (Figure 5E). In accord with this result, IL-8 stimulated the self-renewal capacity of CD44^hi^ IPF MPCs but had only a minimal effect on the self-renewal of CD44^lo^ IPF MPCs (Figure 5F, left and right panels). We next examined the effect of the CXCR1/2 inhibitor Reparixin on the IL-8–mediated increase in Oct4, Sox2, and Nanog expression and self-renewal of CD44^hi^ IPF MPCs. Inhibition of CXCR1/2 function with Reparixin attenuated the IL-8–mediated increase in Oct3/4 expression and completely abrogated the IL-8–mediated increase in Sox2 expression (Figure 5G). No appreciable effect was seen on Nanog expression. Importantly, Reparixin decreased the IL-8–mediated increase in CD44^hi^ IPF MPC self-renewal (Figure 5G). Because IL-8 had the most robust effect on Sox2 gene expression, for the remainder of the experiments we focused on elucidating the mechanism by which IL-8 increases Sox2 gene expression in CD44^hi^ IPF MPCs.

### IL-8 increases CD44 expression and nuclear accumulation.

**Figure 6:**
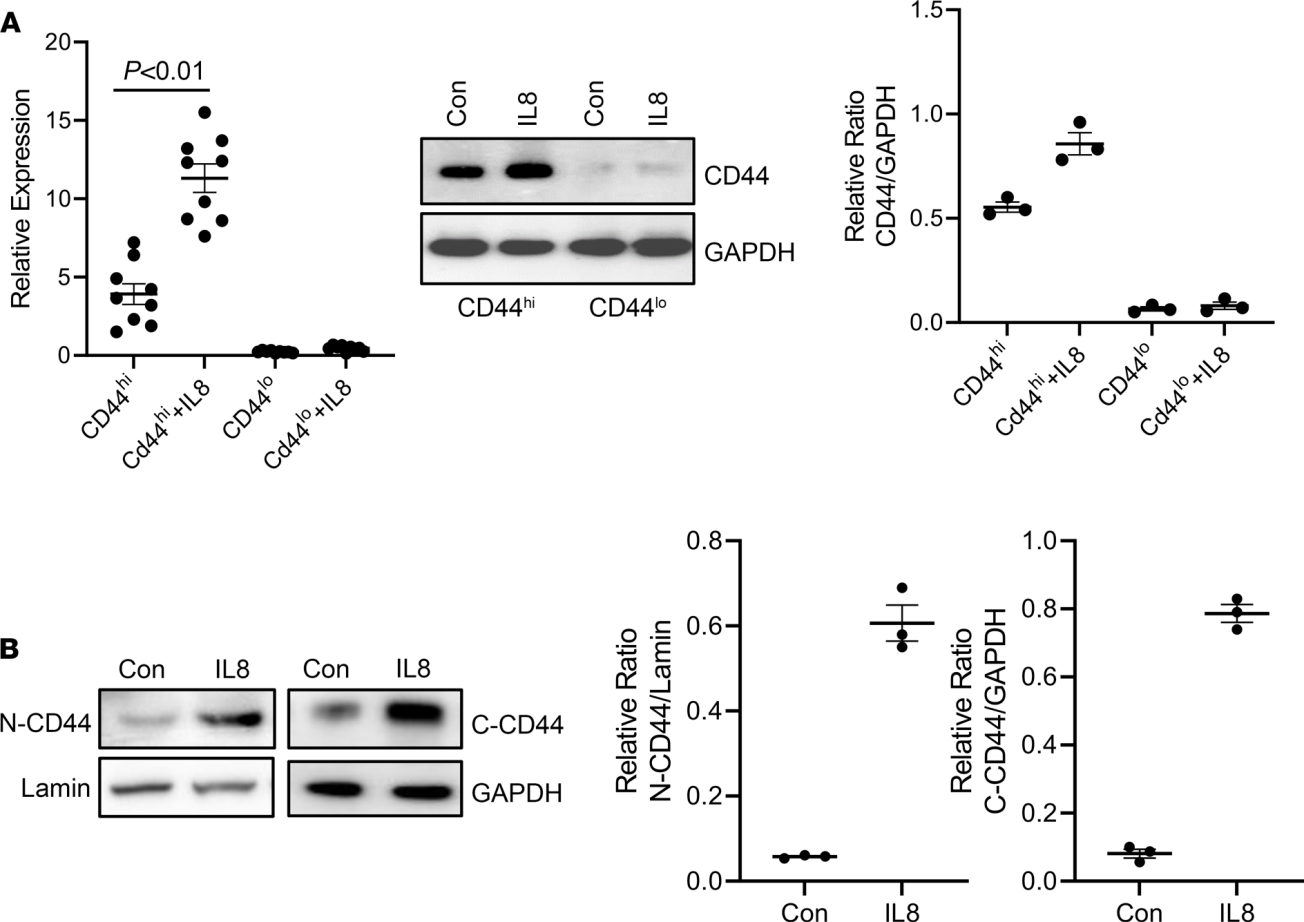
IL-8 increases CD44 expression and nuclear accumulation. (**A**) CD44^hi^ ^and^ ^lo^ IPF MPCs were treated with recombinant IL-8 (5 ng/mL). CD44 mRNA (left panel) and protein (middle panel) expression were quantified by qPCR and Western blot analysis, respectively. Densitometry values summarizing Western blot data in right panel. GAPDH served as loading control. (**B**) CD44^hi^ IPF MPCs were treated with recombinant IL-8 (5 ng/mL). CD44 protein levels were quantified in nuclear (N) and cytoplasmic (C) fractions by Western blot analysis. Lamin (nuclear) and GAPDH (cytoplasmic) served as loading controls. Densitometry values summarizing Western blot data shown in right graphs. IPF 422, IPF424, and IPF 442 were used in these figures. Data are expressed as mean ± SEM. *n* ≥ 3 independent experiments for each experimental condition or group except the Western blot is from a single experiment representative of 3 independent replicates. Data are expressed as mean ± SEM. *P* value in **A** was determined by 2-tailed Student’s *t* test.

Our in vitro and in vivo studies indicate that CD44 plays a mechanistic role in the expression of pluripotency markers, self-renewal, and fibrogenicity of CD44^hi^ IPF MPCs. Since IL-8 potently stimulates CD44^hi^ IPF MPC self-renewal and expression of Sox2, we next examined the role of CD44 in these processes. We first examined the effect of IL-8 on CD44 expression in CD44^hi^ IPF MPCs. Interestingly, IL-8 further increased the mRNA and protein expression of CD44 in CD44^hi^ IPF MPCs (Figure 6A), but exerted only a modest effect on CD44 expression in CD44^lo^ IPF MPCs. Prior studies indicate that full-length CD44 can transit to the nucleus where it plays a role in cancer stem cell colony formation (16–19). Therefore, we next examined the effect of IL-8 on CD44 levels in nuclear and cytoplasmic fractions of CD44^hi^ IPF MPCs. Compared with control, IL-8 markedly increased full-length CD44 nuclear protein levels (83% increase) (Figure 6B).

### Inhibition of CD44 nuclear accumulation blocks IL-8–mediated self-renewal.

**Figure 7:**
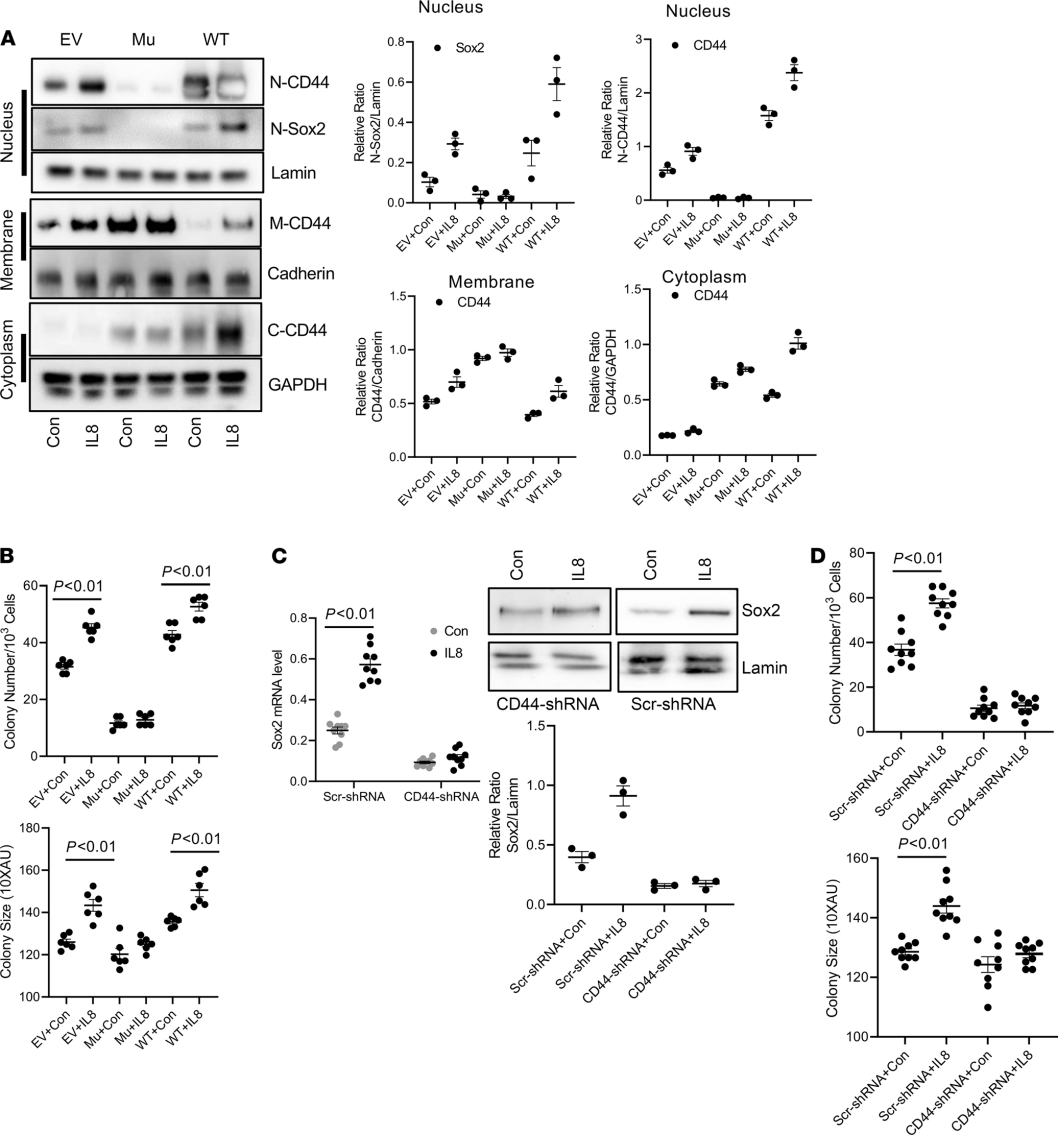
Inhibition of CD44 nuclear accumulation inhibits IL-8–mediated self-renewal. (**A**) CD44^hi^ IPF MPCs transduced with CD44 mutant NLS construct (Mu), WT CD44, or empty vector (EV) were treated with recombinant IL-8 (5 ng/mL) or vehicle control (Con). Sox2 protein expression and nuclear (N), membrane (M), and cytoplasmic (C) CD44 levels were quantified by Western Blot analysis (left panel). Lamin, Cadherin, and GAPDH served as loading controls for N, M, and C fractions, respectively. Densitometry values summarizing Western blot data in right panels. (**B**) CD44^hi^ IPF MPCs transduced with CD44 Mu, WT CD44, or EV were incorporated into methylcellulose gels and treated with IL-8 (5 ng/mL) or vehicle control (Con). Self-renewal was assessed in the colony-forming assay. (**C** and **D**) CD44 was knocked down in CD44^hi^ IPF MPCs using CD44 shRNA (CD44-shRNA). Scrambled shRNA (Scr-shRNA) served as control. Cells were treated with recombinant IL-8 (5 ng/mL) or Con. Shown are Sox2 expression quantified by qPCR (left panel) and Western Blot analysis (right panels). Densitometry values summarizing Western blot data in graph below (**C**). Self-renewal was assessed using the colony-forming assay (**D**). For **C** and **D**, IPF 422, IPF424, and IPF 442 were used in these figures. Data are expressed as mean ± SEM. *n* ≥ 3 independent experiments for each experimental condition or group except the Western blot is from a single experiment representative of 3 independent replicates. Data are expressed as mean ± SEM. *P* values were determined by 2-tailed Student’s *t* test.

To examine the role of nuclear CD44 on IPF MPC self-renewal in response to IL-8, we employed a CD44 mutant construct in which the nuclear localization sequence (NLS) ^292^RRRCGQKKK^300^ had been mutated. Prior studies have shown that when the mutant CD44 NLS construct (^292^AAACGQAAA^300^) becomes engaged, it is internalized into the cytoplasm, but fails to enter the nucleus (16). Consistent with this, CD44 nuclear levels did not increase in CD44^hi^ IPF MPCs transduced with the mutant construct and stimulated with IL-8 (Figure 7A). However, high levels of CD44 were observed in membrane fractions of cells transduced with the mutant construct. In contrast, large amounts of full-length CD44 accumulated in the nucleus of cells transduced with WT CD44 and treated with IL-8 (42% higher in IL-8–treated cells compared with control) (Figure 7A). We therefore examined Sox2 expression in IL-8–treated CD44^hi^ IPF MPCs transduced with the mutant CD44 construct, WT CD44, or empty vector. Sox2 expression was below the limits of detection in cells transduced with the CD44 mutant construct (Figure 7A). However, in response to IL-8 treatment, Sox2 levels increased 66% in cells transduced with WT CD44 compared with cells treated with vehicle control. In contrast, there was no significant difference in Sox2 levels in cells transduced with empty vector and treated with IL-8 compared with control. Consistent with these results, CD44^hi^ IPF MPC self-renewal in response to IL-8 was blunted in cells expressing the mutant construct compared with cells expressing empty vector (Figure 7B). Of note, IL-8 increased CD44^hi^ IPF MPC self-renewal in cells expressing the CD44 WT construct compared with empty vector.

To confirm these results we performed companion studies in which CD44 was knocked down in CD44^hi^ IPF MPCs using CD44 shRNA. Knockdown of CD44 markedly decreased Sox2 expression (73% decrease) in response to IL-8 compared with cells transduced with scrambled shRNA (Figure 7C) and decreased CD44^hi^ IPF MPC self-renewal (Figure 7D). Together, these studies establish a role for nuclear CD44 in regulating Sox2 expression and CD44^hi^ IPF MPC self-renewal induced by IL-8.

### IL-8 promotes the formation of a nuclear CD44/Brg1/Zeb1 protein complex that regulates CD44^hi^ IPF MPC self-renewal.

**Figure 8:**
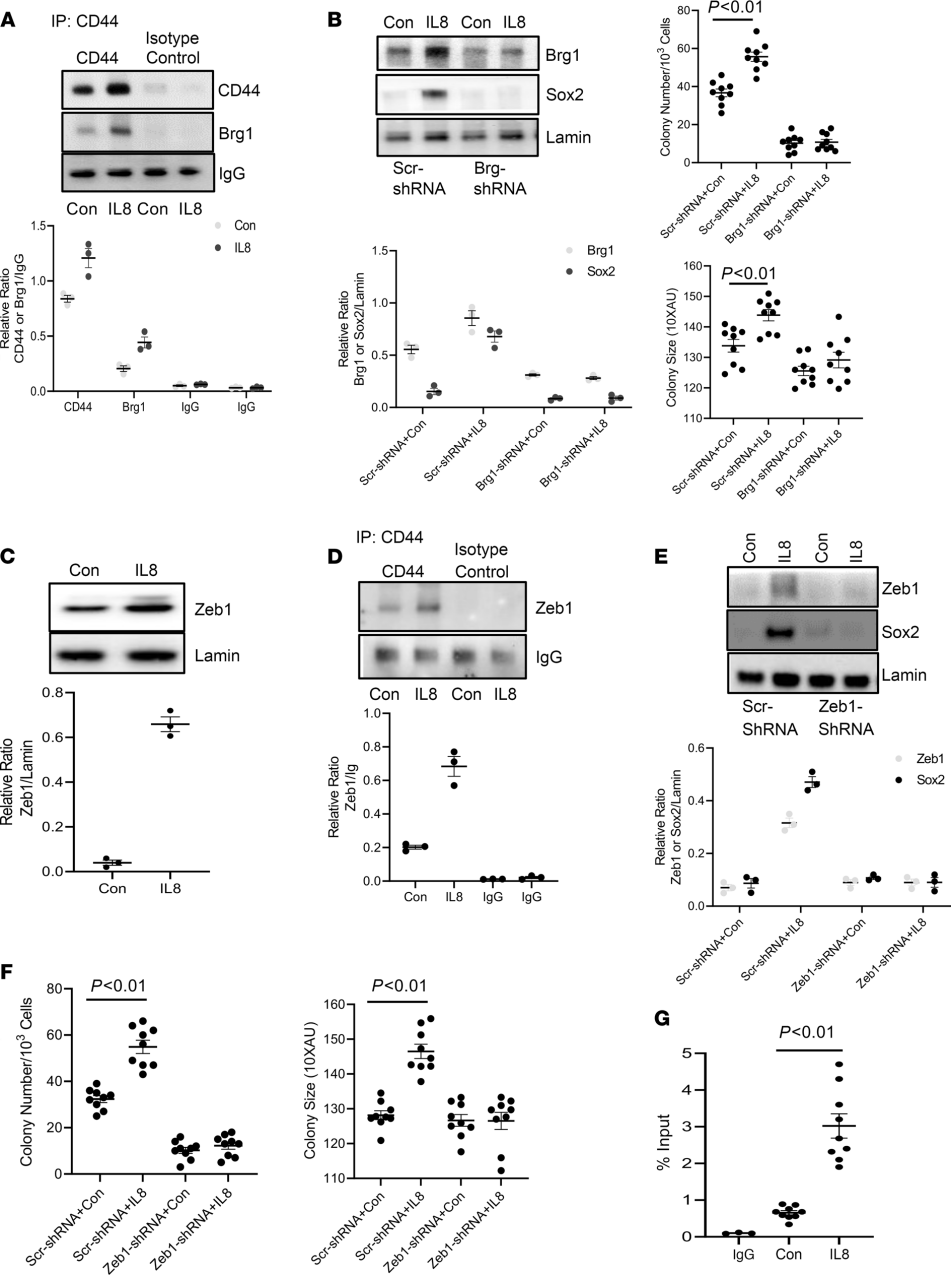
IL-8 promotes the formation of a nuclear CD44/BRG1/Zeb1 protein complex that regulates CD44^hi^ IPF MPC self-renewal. (**A**) CD44^hi^ IPF MPCs were treated with IL-8 or vehicle (Con). Nuclear CD44 was immunoprecipitated, and Western blot analysis for CD44 and BRG1 was performed. Immunoprecipitation using isotype antibody served as control. Densitometry values below Western blot. (**B**) BRG1 was knocked down in CD44^hi^ IPF MPCs using BRG1 shRNA (BRG1-shRNA). Scrambled shRNA (Scr-shRNA) served as control. Cells were treated with IL-8 or vehicle (Con). Sox2 expression was quantified by Western blot analysis (left panel). Densitometry values in graph below. Self-renewal was quantified using the colony-forming assay (right panels). (**C**) CD44^hi^ IPF MPCs were treated with IL-8 or vehicle (Con). Zeb1 protein levels were quantified by Western blot analysis. Densitometry values in graph below. (**D**) CD44^hi^ IPF MPCs were treated with IL-8 or vehicle (Con). Nuclear CD44 was immunoprecipitated, and Western blot analysis for Zeb1 was performed. Immunoprecipitation using isotype antibody served as control. Densitometry values in graph below. (**E** and **F**) Zeb1 was knocked down in CD44^hi^ IPF MPCs using Zeb1 shRNA. Scrambled shRNA (Scr-shRNA) served as control. The cells were treated with IL-8 or vehicle (Con). Zeb1 and Sox2 expression were quantified by Western blot analysis (densitometry values in graph below) (**E**), and self-renewal was quantified using the colony-forming assay (**F**). (**G**) CD44^hi^ IPF MPCs were treated with IL-8 or vehicle (Con). Zeb1 was immunoprecipitated from nuclear fractions, and qPCR for Sox2 was performed. Immunoprecipitation using isotype antibody (IgG) served as control. IPF 422, 424, and 442 were used in these figures. Data are expressed as mean ± SEM. *n* ≥ 3 independent experiments for each experimental condition, except the Western blot, which is from a single experiment representative of 3 independent replicates. Data are expressed as mean ± SEM. *P* values were determined by 2-tailed Student’s *t* test.

To elucidate the mechanism by which nuclear CD44 regulates IL-8–mediated CD44^hi^ IPF MPC self-renewal, we subjected the nuclear CD44 interactome to proteomic analysis. We treated CD44^hi^ IPF MPCs with IL-8 and immunoprecipitated nuclear CD44. The proteomic analysis revealed that nuclear CD44 is in a protein complex with Brg1 (Supplemental Table 1). Brg1 is an ATPase subunit within the Switch/Sucrose Nonfermentable (SWI/SNF) chromatin-remodeling complex functioning as an epigenetic modulator protein (20–23). To confirm the mass spectroscopy results, CD44^hi^ IPF MPCs were treated with IL-8. CD44 was immunoprecipitated from nuclear fractions and Western blot analysis for Brg1 was performed. Consistent with our findings that IL-8 increases nuclear CD44 levels, we found that compared with control, IL-8 also promoted the interaction of nuclear CD44 with Brg1 (Figure 8A). To analyze the role of Brg1 in the process, we performed a Brg1 loss of function experiment. Knockdown of Brg1 inhibited IL-8–mediated Sox2 expression (Figure 8B) and CD44^hi^ IPF MPC self-renewal (Figure 8B). These data support the concept that IL-8 promotes the formation of a CD44/Brg1 nuclear protein complex that mediates the IL-8 induced increases in Sox2 expression and CD44^hi^ IPF MPC self-renewal.

SWI/SNF complexes containing Brg1 are typically recruited to genomic sites via their interaction with other proteins including sequence specific zinc finger transcription factors (20). Since our data indicate that the CD44/Brg1 nuclear protein complex regulates Sox2 expression, this suggests that the CD44/Brg1 complex may require interaction with specific transcription factor(s) in order to regulate Sox2 expression. Our proteomics analysis also identified Zeb1 as a candidate component of the nuclear CD44 interactome and a prior study determined that Brg1 can associate with the Zeb1 transcription factor (24) (Supplemental Table 1). Based on this information, we first examined the effect of IL-8 on Zeb1 expression in CD44^hi^ IPF MPCs. IL-8 increased Zeb1 expression 90% compared with vehicle control (Figure 8C). To determine whether Zeb1 forms a protein complex with CD44/Brg1 in response to IL-8, we immunoprecipitated nuclear CD44 and performed Western blot analysis for Zeb1. IL-8 treatment promoted the interaction of Zeb1 with nuclear CD44 (Figure 8D). Based on these results, we further examined whether Zeb1 regulates the increase in Sox2 expression in CD44^hi^ IPF MPCs in response to IL-8. Knockdown of Zeb1 inhibited the IL-8–mediated increase in Sox2 expression (Figure 8E). We next examined whether Zeb1 knockdown was required for IL-8–mediated IPF MPC self-renewal. Knockdown of Zeb1 inhibited the IL-8–mediated increase in colony formation (Figure 8F). We next sought to determine if the CD44/Brg1 complex containing Zeb1 directly targets the Sox2 gene by ChIP analysis. CD44^hi^ IPF MPCs were treated with IL-8, Zeb1 was immunoprecipitated and PCR for Sox2 was performed. ChIP analysis indicated that Zeb1 directly interacts with the Sox2 promoter (Figure 8G). Taken together, our data indicate that in response to IL-8, a nuclear CD44/Brg1/Zeb1 complex forms that directly targets the Sox2 gene increasing its expression and promoting CD44^hi^ IPF MPC self-renewal.

## Discussion

IPF is an unrelenting, progressive fibrotic lung disease. Identification of the obligatory drivers of fibrotic progression will be required to develop effective treatments that arrest the disease process. We have previously identified IPF MPCs in the lungs of patients with IPF that serve as a source for IPF fibroblasts (4). These cells express the progenitor marker SSEA4, are found concentrated on the periphery of fibroblastic foci in human IPF lung tissue, and display cell-autonomous fibrogenicity, producing nonresolving interstitial lung disease in a humanized mouse xenograft model (5). Our recent analysis of IPF MPCs by single-cell RNA-Seq demonstrated heterogeneity within the IPF MPC population (10). Among the entire population of IPF lung MPCs, the least differentiated cells (highest transcriptional network entropy) were those that displayed the greatest differences in their transcriptome compared with lung MPCs from patient controls. These data indicate that IPF MPCs acquire a cell-autonomous pathological phenotype early in their differentiation trajectory. Importantly, we identified CD44 as a marker of these high entropy MPCs, suggesting that IPF MPCs with CD44^hi^ expression may display enhanced fibrogenicity. Here we show that CD44^hi^ IPF MPCs display greater self-renewal capacity and are more fibrogenic than CD44^lo^ IPF MPCs in humanized mice. The increased proliferative capacity of CD44^hi^ IPF MPCs compared with CD44^lo^ IPF MPCs is likely responsible for the increased numbers of CD44^hi^ IPF MPCs present in the lungs of mice receiving these cells as demonstrated in the adoptive transfer mouse model. Knockdown of CD44 diminished their fibrogenicity indicating that CD44 plays a mechanistic role in their fibrogenic phenotype. We have found that the increased expression of stemness markers and greater self-renewal capacity of CD44^hi^ IPF MPCs compared with CD44^lo^ IPF MPCs is potentiated by IL-8. CD44^hi^ IPF MPCs express higher levels of the IL-8 receptor CXCR1 and secrete more IL-8 compared with CD44^lo^ IPF MPCs. IL-8 promotes the accumulation of CD44 in the nucleus where it forms a protein complex with Brg1 and the Zeb1 transcription factor. This complex directly increases the expression of several stem cell markers including Sox2 and Oct3/4. Importantly, IHC analysis of IPF lung tissue revealed that CD44-expressing MPCs are concentrated at the periphery of the fibroblastic focus, placing these cells in regions of active fibrogenesis. Our data identify CD44 at the apex of a pathway conferring IPF MPCs with robust fibrogenicity.

The cell surface receptor CD44 is an important regulator of stem cell self-renewal (18). Not only do normal stem cells express high levels of CD44, but CD44 is also highly expressed by cancer cells and serves as a cell surface marker facilitating their isolation (25–27). Similarly, our single-cell sequencing study showed that CD44 was a marker of the least-differentiated IPF MPCs as assessed by transcriptional entropy. In addition to serving as a marker of transcriptional entropy in fibrogenic MPCs, our data indicate that CD44 plays an important role in conferring these cells with fibrogenicity. In accord with this, genes correlated with CD44 expression are related to fibrotic functions (Supplemental Table 2). Toward the top of the list is PTEN, an inhibitor of the PI3K/Akt pathway that we have previously shown to be important in regulating IPF fibroblast proliferation and survival (28–30). Interestingly, SUMO2 is also included in the list. SUMO molecules are important in promoting sumoylation of S100A4 and its nuclear localization. We have previously shown that S100A4 is a key regulator of IPF MPC self-renewal (5). Of potential relevance to the current study identifying CD44/Brg1 nuclear complex in regulating IPF MPC self-renewal, several RNA splicing factors (e.g., SRSF6 & SRSF3) which have been identified as target genes for Brg1 are included in the list of top 20 genes correlated with CD44 expression. Our data indicate that these cells have high levels of full-length CD44 within their nucleus. Prior studies have demonstrated that when the CD44 receptor is ligated, full-length CD44 is internalized and transported to the nucleus where it regulates cellular functions (16–19). We discovered that IL-8 promotes full-length CD44 nuclear accumulation, a process not previously described. Of note, studies indicate that both full-length CD44 and an intracellular cytoplasmic domain (ICD) fragment of CD44 can transit to the nucleus where it participates in nuclear signaling events. While studies indicate that proteolysis of the CD44 extracellular domain leads to the release and internalization of CD44 ICD (31, 32), the mechanism by which full-length CD44 is internalized remains unclear. The primary isoform of CD44 found in the nucleus of IPF MPCs treated with IL-8 was full-length CD44. Likewise, we found that large amounts of full-length CD44 accumulated in the nucleus of IPF MPCs transduced with WT CD44 and our data indicate that full-length CD44 associates with Brg1 in response to IL-8 treatment. Together, these data support the concept that a CD44/Brg1 complex participates in nuclear signaling events in IPF MPCs stimulated with IL-8.

A prior study examining breast cancer stem cells demonstrated that these cells express high levels of the IL-8 receptor CXCR1 and that IL-8 promotes cancer stem cell self-renewal. Similar to cancer stem cells, we found that CD44^hi^ IPF MPCs also express high levels of CXCR1 and show that IL-8 increases the expression of stemness markers Sox2 and Oct3/4 and promotes their self-renewal. We would like to emphasize that while IL-8 promoted CD44 nuclear accumulation in IPF MPCs and is an important exogenous cue in the IPF fibrogenic niche (9), other exogenous cues are also likely to regulate IPF MPC function. For example, the CD44-activating antibody Hermes 3 promotes CD44 nuclear translocation, suggesting that exogenous cognate ligands such as hyaluronan binding to the CD44 receptor may promote CD44 nuclear accumulation. We view IL-8 as likely being one of many exogenous ligands that promote CD44 nuclear translocation and therefore the focus of this study was not on IL-8 per se, but on the role of nuclear CD44 in regulating IPF MPC fibrogenicity. Importantly, we demonstrated that nuclear CD44 plays a key role in these processes. CD44 contains a nuclear localization sequence that facilitates its entry into the nucleus (16, 17). Using a CD44 mutant construct in which we mutated the CD44 NLS sequence, we demonstrate that the IL-8–mediated accumulation of CD44 in the nucleus is inhibited and the increase in stemness marker expression and cell renewal are diminished. In contrast, IPF MPCs transduced with WT CD44 contained abundant nuclear full-length CD44 and displayed increased self-renewal capacity. Of note, it has been demonstrated that CD44 nuclear import is dependent upon its interaction with transportin1 and while mutation of the CD44 nuclear localization sequence impairs CD44 nuclear import, the CD44 NLS mutant is still capable of binding transportin1 (16). On the basis of this knowledge, we suspect that the ability of the CD44 NLS mutant to inhibit CD44 nuclear accumulation, including endogenous CD44 entry into the nucleus in response to IL-8, is due to the ability of the mutant construct to sequester transportin1 in the cytoplasm. In this regard, a prior publication has demonstrated that the CD44 NLS mutant is capable of sequestering certain transcription factors in the cytoplasm, preventing their entry into the nucleus (17). Together, these data suggest that within the fibrogenic niche of the IPF lung, these highly entropic CXCR1 expressing CD44^hi^ IPF MPCs are primed to undergo self-renewal in response to IL-8. In this regard, in a prior study, we found IPF MPCs at the periphery of the fibroblastic focus co-distributing with activated macrophages, a potential source of cytokines. This raises the possibility that macrophage cross-talk with MPCs may augment their fibrogenic function (9). Under this scenario, residence in the fibrogenic niche could promote expansion of the fibrogenic CD44^hi^ IPF MPC population and serve to drive progression of fibrosis. Thus, as in cancer progression, a strategy targeting cell-cell and cell-ECM interactions may be necessary to effectively halt IPF fibrotic progression.

To begin to elucidate the mechanism by which nuclear CD44 regulates the expression of stemness markers and self-renewal, we performed an unbiased proteomic analysis of nuclear CD44 immunoprecipitated from CD44^hi^ IPF MPCs treated with IL-8 to define the nuclear CD44 interactome. The proteomic analysis revealed that CD44 forms a protein complex with Brg1 (also termed SMARCA4) within the nucleus, a finding confirmed by immunoprecipitation studies. We further demonstrate that IL-8 increases the formation of CD44/Brg1 nuclear complexes. Brg1 is an ATPase subunit within the SWI/SNF chromatin-remodeling complex (20–23). Brg1/SWI/SNF complexes are typically recruited to genomic sites via their interaction with other proteins including sequence-specific zinc finger transcription factors (20). Once recruited to a genomic site, Brg1 regulates gene expression using the energy derived from ATP hydrolysis to disrupt histone-DNA interactions (33). Recent work indicates that Brg1 (SMARCA4) regulates higher-order chromatin structure and may regulate gene expression by altering local chromatin accessibility around target gene sites (34). Importantly, Brg1 binding is concentrated at topologically-associating domains (TADs), where specific long-range chromatin looping interactions occur. This interaction of Brg1 at TAD sites is thought to regulate chromatin structures, thereby affecting the function of specific transcription factors by altering transcription factor recruitment and promoter occupancy (35). Interestingly, while initially thought to lack DNA specificity, recent evidence suggests that Brg1 has DNA-binding activity with specificity for AT-rich elements (36). Under physiologic conditions, Brg1 regulates a variety of cellular processes including stem/progenitor cell self-renewal and the response to hypoxia (22, 23). Importantly, abnormalities in Brg1 have been linked to disease pathogenesis. Brg1 plays a dual role in cancer. It is mutated in approximately 20% of cancers where its silencing supports cancer initiation (37, 38), and high levels of Brg1 are associated with cancer progression (39–41). Consistent with its ability to modify chromatin structure, Brg1 is a key epigenetic regulator of cancer cells (41–43). Of potential relevance to the lung fibrosis in IPF, Brg1 expression is increased in human liver fibrosis and experimental models of liver fibrosis. Elevated expression of Brg1 promotes liver fibrosis by a mechanism involving activation of hepatic stellate cells — a precursor cell that gives rise to myofibroblasts in liver fibrosis (44).

Taken together, these data raise the possibility that the CD44/Brg1 nuclear complex functions by generating epigenetic alterations in the chromatin structure of IPF MPCs, thereby affecting the function of specific transcription factors that underlie their heightened fibrogenicity in response to key external cues such as cytokine exposure. In support of this hypothesis, we demonstrate that CD44 and Brg1 are required for the IL-8–mediated increase in stemness marker expression and CD44^hi^ IPF MPC self-renewal. Since the CD44/Brg1 complex promotes stemness marker expression, and Brg1 associates with specific transcription factors that facilitate Brg1 DNA targeting, we sought to determine whether the CD44/Brg1 nuclear complex interacts with specific transcription factors that regulate the expression of stemness markers. Our proteomic analysis to elucidate the nuclear CD44 interactome identified Zeb1 as a candidate transcription factor that may interact with the CD44/Brg1 complex and regulate progenitor transcription factor expression. This is consistent with a previous study demonstrating Brg1 association with Zeb1 transcription factor (22). Furthermore, Zeb1 has been shown to be a crucial regulator for acquisition of EMT-like and stemness phenotypes in cancer cells, with the mechanism featuring a self-enforcing CD44s/Zeb1 feedback loop (24, 45–47). Here we demonstrate that IL-8 greatly increased the expression of Zeb1 in CD44^hi^ IPF MPCs. Immunoprecipitation studies indicated that the interaction of Zeb1 with nuclear CD44 was enhanced by IL-8. Follow-on ChIP-PCR studies showed that Zeb1 directly associates with the Sox2 promoter in response to IL-8. Consistent with this, Zeb1 knockdown functional studies showed that Zeb1 is required for Sox2 expression and CD44^hi^ IPF MPC colony formation in response to IL-8.

While our studies demonstrate a role for the CD44/Brg1/Zeb1 complex in regulating Sox2, prior studies have shown that Brg1 can interact with a variety of transcription factors including the repressor element 1-silencing transcription factor (REST) and Sp1 (24, 48, 49). This raises the possibility that the mechanism by which the CD44/Brg1 nuclear complex regulates IPF MPC self-renewal may involve interactions with other transcription factors in addition to Zeb1, a focus of ongoing work in our laboratory. Furthermore, it is important to note that we focused on the Sox2 as an exemplar progenitor-transcription factor whose expression was most responsive to IL-8 treatment in CD44^hi^ IPF MPCs. However, in addition to binding the Sox2 promoter, the Zeb1 transcriptional program includes the Oct3/4 promoter, a stemness factor whose expression was also increased in CD44^hi^ IPF MPCs by IL-8 (50).

In summary, this work identifies a CD44-initiated transcriptional program involving Brg1 and Zeb1 that conveys IPF MPCs with cell-autonomous fibrogenicity and heightened sensitivity to IL-8 in IPF.

## Methods

### Primary mesenchymal cell lines.

Six primary lung mesenchymal cell lines were established from 6 individual patients fulfilling diagnostic criteria for IPF including a pathological diagnosis of usual interstitial pneumonia (51). Cell lines were derived from lungs, characterized as mesenchymal cells, and cultivated as previously described (4, 5).

### Isolation of mesenchymal progenitor cells.

For isolation of CD44^hi^ ^and^ ^lo^ IPF MPCs, primary IPF mesenchymal cells were labeled with mouse anti-human SSEA4 antibody conjugated to Alexa Fluor 647 (BD Biosciences, clone MC-813-70, catalog 560796) and mouse anti-human CD44 conjugated to FITC (BioLegend, clone IM7, catalog 103006). Cells were sorted on a FACS Aria Cell Sorter (BD Biosciences). Cells that were SSEA4+ and CD44+ (relative to mouse IgG3 κ isotype control conjugated to Alexa Fluor 647 and mouse IgM κ isotype control conjugated to FITC, respectively) (BD Biosciences, clone J606, catalog 560803; BioLegend, catalog 402207) were collected as we previously described (14). For CD44^hi^ IPF MPC isolation, the FACS Sorter gate was set to collect SSEA4 positive cells at the top 3% of CD44 expression and for CD44^lo^ IPF MPCs, the collecting gate was set to collect SSEA4 positive cells at the bottom 3% of CD44 expression. To generate sufficient numbers of MPCs for the in vivo mouse studies, SSEA4+ cells were expanded by culture in DMEM + 10% FCS for 7 days prior to use. The resulting MPC cultures were reanalyzed for SSEA4 expression by FAC analysis and for colony formation in vitro. 97% of day 7 MPCs were SSEA4 positive and formed colonies in methylcellulose, indicating retention of progenitor self-renewal properties.

### qPCR.

Analysis of CD44, CXCL8, CXCR1, CXCR2, Sox2, Nanog, OCT4, and Zeb1 gene expression was conducted by qPCR as previously described (9). Total RNA was isolated and reverse transcribed using a Taqman Reverse Transcriptase Reagent Kit (Roche) and primed with random hexamers. Primer sequences were selected using NCBI Primer-BLAST. Real-time PCR (qPCR) was performed using a LightCycler FastStart DNA Master SYBR Green I Kit (Roche). Primer sequences were as follows: GAPDH forward, 5′-TGTTGCCATCAATGACCCCTT-3′; GAPDH reverse, 5′-CTCCACGACGTACTCAGCG-3′; CD44 forward, 5′-GCTACCAGAGACCAAGACACA-3′; CD44 reverse, 5′-GCTCCACCTTCTTGACTCC-3′; CXCL8 forward, 5′-cttggcagccttcctgattt-3′; CXCL8 reverse, 5′-ttctttagcactccttggcaaaa-3′; CXCR1 forward, 5’ TGGGGACTGTCTATGAATCTGT-3’; CXCR1 reverse, 5’-GCAACACCATCCGCCATTTT-3’; CXCR2 forward, 5′-CACCGATGTCTACCTGCTGA-3′; CXCR2 reverse, 5′-CACAGGGTTGAGCCAAAA GT-3′; Sox2 forward, 5′-GGGAAATGGGAGGGGTGAAAAGAGG-3’; Sox2 reverse, TTGCGTGAGTGTGGATGGGATTGGTG-3’; Nanog forward, 5′-TGTCTTCTGCTGAGATGCCTCACA-3’; Nanog reverse, 5′-CCTTCTGCGTCACACCATTGCTAT-3’; OCT4 forward, 5′-GACAACAATGAGAACCTTCAGGAG A-3′; OCT4 reverse, 5′ CTGGCGCCGGTTACAGAACCA-3′; Zeb1 forward, 5′-GCCAACAGACCAGACAGTGTT-3’; Zeb1 reverse, 5′-TTTCTTGCCCTTCCTTTCTG-3’; Sox2 promoter forward, 5′-ggggtaccGGGGGGAGTGCTGTGGATGAG-3’; and Sox2 promoter reverse, 5′-cccaagcttGCCTGGGGCTCAAACTTCTCT-3’.

Samples were quantified at the log-linear portion of the curve using LightCycler analysis software (Roche) and compared with an external calibration standard curve.

### Self-renewal assay.

Single-cell suspensions of CD44^hi^ ^and^ ^lo^ IPF MPCs were incorporated into methylcellulose gels (Stemcell Technologies) and maintained in MSC SFM CTS (Gibco, Thermo Fisher Scientific) (37°C, 5% CO_2_; 1 week). Enumeration of colonies was performed microscopically and the colony size was quantified by ImageJ. In some self-renewal assays the cells were treated with the indicated concentrations of recombinant IL-8 (R&D Systems).

### IL-8.

IL-8 secretion was quantified in CD44^hi^ ^and^ ^lo^ IPF MPC conditioned media by ELISA as previously described (9).

### Mass spectrometry.

Nuclear fractions of IPF MPCs were isolated by NE-PER Nuclear and Cytoplasmic Extraction reagents (Thermo Fisher Scientific). CD44 was immunoprecipitated from the resulting nuclear lysates using a CD44 antibody (2C5, R&D Systems). Control consisted of immunoprecipitation using an isotype control antibody. The immunoprecipitates were subjected to SDS-PAGE followed by silver staining (Pierce Silver Stain Kit, Thermo Fisher Scientific). Bands were excised, destained, and subjected to in-gel trypsin digestion. Peptide samples were resuspended in 98:2 water/acetonitrile, 0.1% formic acid and run on an Orbitrap Velos (Thermo Fisher Scientific) mass spectrometer. The Thermo RAW files were analyzed with PeaksStudio 7.0 build 20140912 (Bioinformatics Solutions) software package for interpretation of tandem mass spectrometry and protein inference.

### Plasmids/constructs.

For loss of function, CD44, Brg1, and Zeb1 were knocked down using shRNA (pGIPZ-CD44, pGIPZ-Brg1, or pGIPZ-Zeb1 shRNA; IDT and UMN Genomics Center). Scrambled shRNA served as control. The CD44 nuclear localization sequence mutant was constructed by site-directed mutagenesis using WT CD44S isoform cDNA as a template as previously described (17). The putative nuclear localization sequence ^292^RRCGQKKK^300^ was changed to ^292^AAACGQAAA^300^. The mutant constructs were verified by DNA-Seq. Cells were transduced using a lentiviral vector containing mutant, WT, or empty vector constructs.

### Western blot and immunoprecipitation.

Western blots were performed as previously described (4–6). For immunoprecipitation, nuclear fractions were isolated by NE-PER Nuclear and Cytoplasmic Extraction reagents. The samples were centrifuged at 12,000*g* for 15 minutes at 4°C, and the lysates were precleared for 1 hour at 4°C with protein A/G beads and immunoprecipitated for 2 hours at 4°C with the appropriate primary antibody.

### ChIP assay.

ChIP Assay kit (Ab185913, Abcam) was used. Six × 10^6^ cells were treated with 5 ng of IL-8 or vehicle control. The experiment was conducted following the manufacturer’s instruction. ChIP-PCR was performed as delineated above.

### IHC of IPF lung tissue.

IHC was performed on 4 μm paraffin-embedded serial-sectioned IPF lung tissue and mounted on polylysine-coated slides. The sections were deparaffinized in xylene, rehydrated through a graded Methanol series, quenched with 0.3% Hydrogen Peroxide in Methanol, and immersed in a 98°C water bath for 30 minutes in Citrate Buffer (pH 6·0) for antigen retrieval. Sections were placed in 5% Normal Horse Serum (Jackson ImmunoResearch) to block nonspecific binding of secondary antibodies. A multiplex IHC kit was used for antigen detection according to the manufacturer’s instructions (MULTIVIEW IHC Kit ADI-950.101.000, Enzo Life Sciences). The tissue specimens were incubated overnight (18–20 hours, 4°C) with the following primary antibodies: anti-rabbit CD44 monoclonal antibody (1:800) (Spring Bioscience, catalog M3370); and anti-human SSEA4 antibody (1:100) (BioLegend, clone MC-813-70, catalog 330402). Specimens were cover-slipped with a Prolong Antifade Kit (Invitrogen Molecular Probes, Thermo Fisher Scientific) and stored overnight at room temperature without light before image analysis.

### Mouse xenograft model of fibrotic progression.

To assess the ability of CD44^hi^ or CD44^lo^ IPF MPCs to drive fibrotic progression in vivo, we utilized our IPF MPC-based mouse model of fibrotic progression (5). NOD/SCID/IL2rγ/B2M (NSG) male and female mice (Jackson Laboratories), average 10 weeks of age, were administered intratracheal bleomycin (1.25 U/kg) to establish self-limited lung fibrosis. Two weeks following bleomycin administration, CD44^hi^ or CD44^lo^ IPF MPCs were suspended in PBS (10^6^ cells/100 μL) and injected via the tail vein following our published protocol (5). Alternatively, to examine the role of CD44 in regulating IPF MPC fibrogenicity, IPF MPCs transduced with CD44 shRNA or scrambled shRNA were administered to the mice. Mice were euthanized 4 weeks after adoptive transfer of human cells and the lungs were harvested. Collagen content was quantified in the left lung tissue by Sircol assay and served as the primary endpoint (4, 5). Histological (H&E and trichrome staining) and IHC analysis were performed on the paraffin-embedded and frozen right lung tissue. Cells positive for human procollagen (anti-human procollagen type I antibody, 1:500; EMD Millipore, catalog MAB1912) were identified as human. The presence of lung fibrotic lesions by histological analysis served as the secondary endpoint. IHC using the human procollagen antibody and CD44 antibody was performed to assess the distribution of CD44-expressing cells with human procollagen I-expressing cells.

### Statistics.

Comparisons of data among experiments were performed using the 2-tailed Student’s *t* test and ordinary 1-way ANOVA as indicated in the figure legend. Data are expressed as mean ± SEM. *P* < 0.05 was considered significant.

### Study approval.

De-identified patient samples were obtained by our tissue procurement service (Bionet) under a waiver of informed consent from the University of Minnesota Institutional Review Board (University of Minnesota IRB ID: 1504M68341). Animal protocols were approved and conducted in accordance with the University of Minnesota Institutional Animal Care and Use Committee regulations (approval 1706-34890A).

### Data and materials availability.

Single-cell RNA-Seq data for IPF and control MPCs deposited in: BioProject repository accession PRJNA641647.

## Author contributions

LY and CAH conceived, designed, and directed the studies with input from PBB and DB. LY, CAH, and PBB wrote the manuscript with assistance from all the authors. LY established the primary human mesenchymal cell lines, cultured MPCs, performed flow cytometry for isolation of MPCs, performed qPCR, performed Western blot analysis, and performed gain- and loss-of-function experiments, mouse studies, and IHC. AG and HX assisted with isolation and culture of MPCs, as well as assisted with mouse studies. HX also assisted with IHC analysis. JK performed IHC analysis. KS designed and constructed expression constructs and performed flow cytometry for isolation of MPCs.

## Supplementary Material

Supplemental data

## Version 1 04/06/2021

In-Press Preview

## Version 2 05/10/2021

Electronic publication
