# Supplementary material for: A CD44/Brg1 nuclear complex confers mesenchymal progenitor cells with enhanced fibrogenicity in idiopathic pulmonary fibrosis
Source: JCI Insight. 2021 May 10;6(9):e144652. doi: 10.1172/jci.insight.144652 (PMC8262361; doi:10.1172/jci.insight.144652)
Supplement: Supplemental data [file jciinsight-6-144652-s279.pdf]

SUPPLEMENTARY FIGURES

Supplementary Figure 1

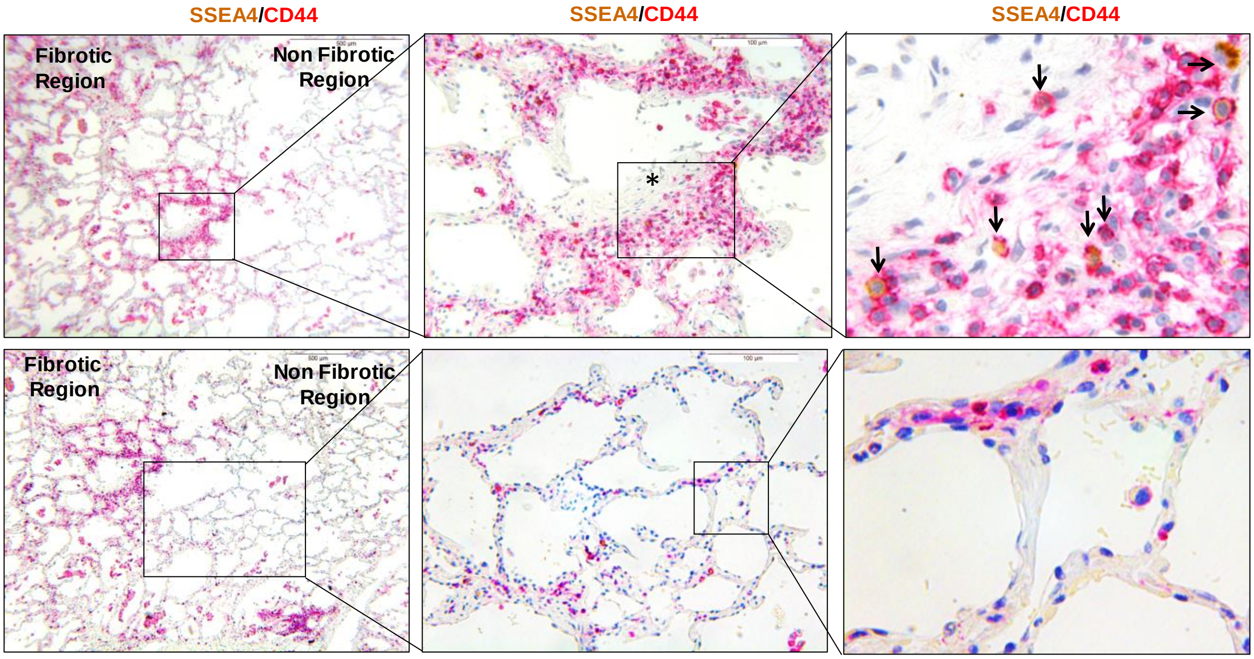

Supplementary Figure 1. Immunohistochemistry double staining for SSEA4 (brown) and CD44 (red) of IPF lung tissue. Shown is a fibroblastic focus at the interface between fibrotic lung tissue and less involved lung tissue. Upper panels: Boxed region displays a fibroblastic focus. Asterisk denotes focus (myofibroblast) core. Upper right hand panel: SSEA4+/CD44+ MPCs are present on the periphery of the fibroblastic focus. Arrows point to double positive cells (SSEA4+/CD44+). Note absence of MPCs (double positive cells) in focus core. Bottom panels: Boxed region denotes a less involved area adjacent to the fibroblastic focus shown in the upper panels. Note a lack of double positive (SSEA4+/CD44+) MPCs in a relatively uninvolved alveolar structure. Scale bars: 500 µm (left hand panels); 100 µm (middle panels).

Supplementary Figure 2

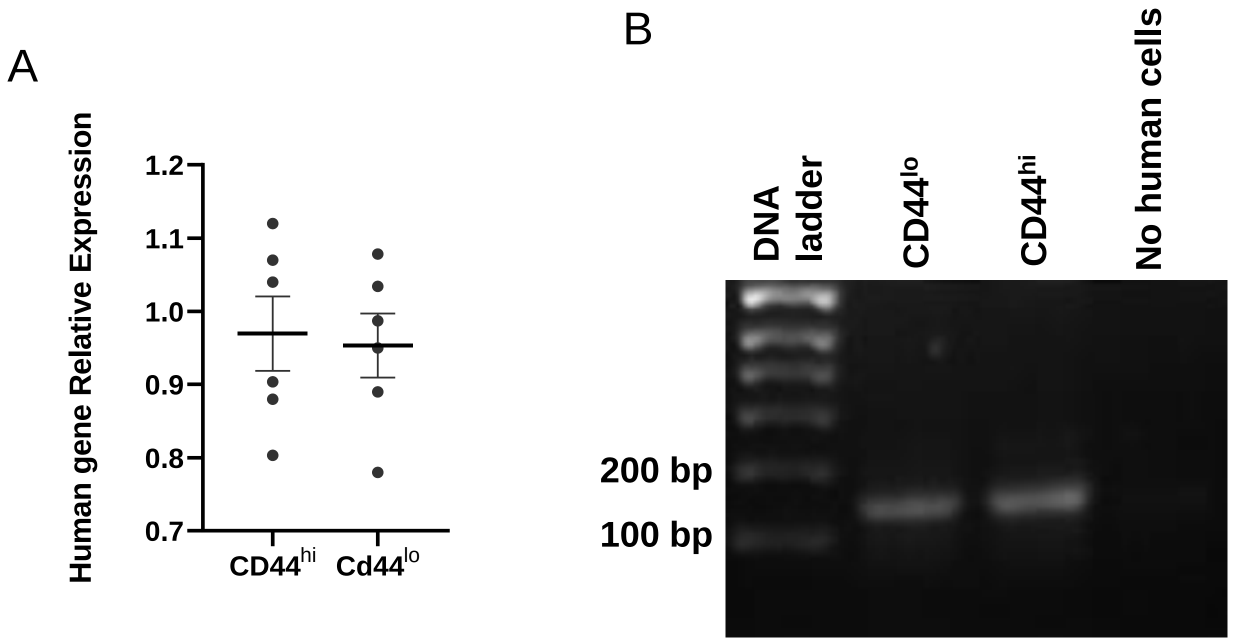

Supplementary Figure 2. Confirmation of engraftment of human CD44$^{hi\ and\ lo}$ IPF MPCs in immune-deficient mice by real-time PCR. Three groups of immune-deficient mice (n=2 each) were administered intratracheal bleomycin (1.25 U/kg). In 2 groups of mice, 10^6 IPF CD44$^{hi\ and\ lo}$ MPCs were injected via tail vein 2 weeks after intratracheal bleomycin. The third group did not receive human cells and served as a negative control. Seven days after administration of cells the lungs were harvested for analysis of engraftment of human cells. The lungs were digested and genomic DNA isolated using a PureLink Genomic DNA Mini Kit according to the manufacturer's instructions (Invitrogen, Carlsbad,CA). Real-time PCR was used to quantify human IPF cells in the mouse lungs by measuring the amount of human-specific DNA sequence using human specific primers per a previously published protocol (38). PCR assay was performed for 40 cycles using the human genomic DNA-specific primers (forward: ATGCTGATGTCTGGGTAGGGTG; reverse: TGAGTCAGGAGCCAGCGTATG). A. Engraftment of human IPF CD44$^{hi\ and\ lo}$ MPCs was quantified by Q-PCR. B. Shown is gel electrophoresis of the PCR products. A single, predicted 141 base pair band specific for the human genomic DNA sequence was amplified from the DNA extracted from the lungs of mice receiving human cells (lanes 3 and 4). Lane 1: DNA ladder. Lane 2: mouse lung containing human CD44^{lo} IPF MPCs. Lane 3: mouse lung containing human CD44^{hi} IPF MPCs. Lane 4: mouse lung only (no human cells).

Supplementary Figure 3

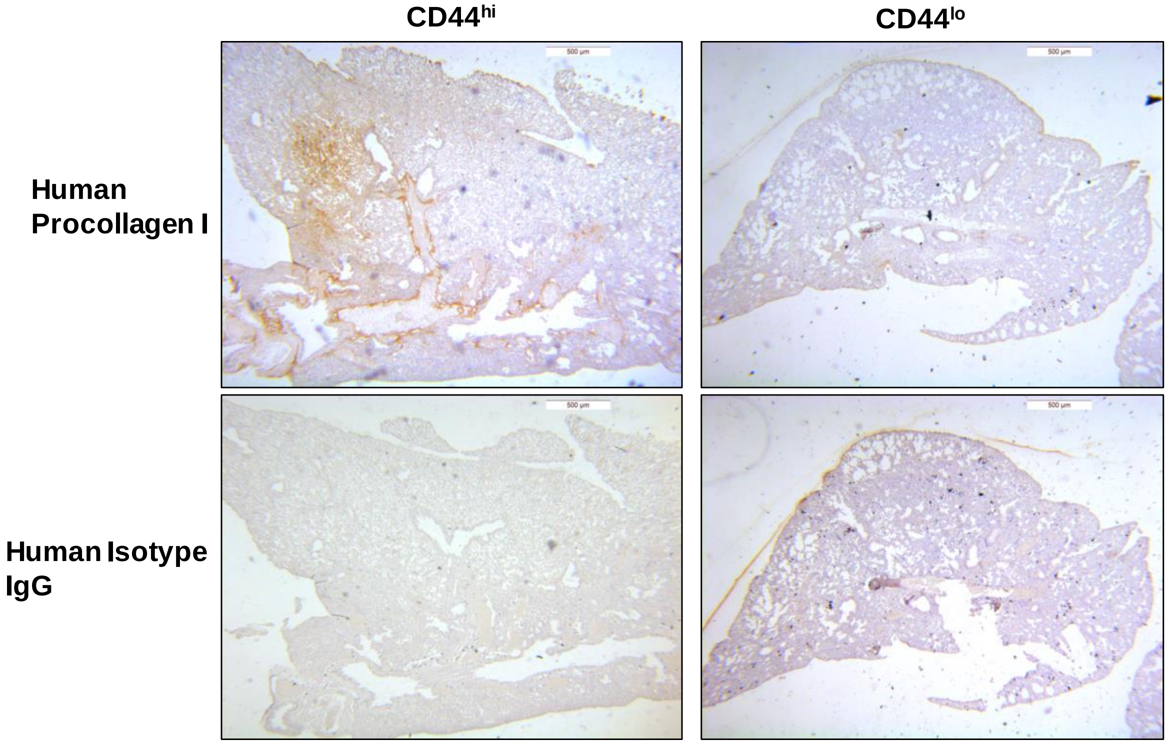

Supplementary Figure 3. Immunohistochemistry of mouse lung tissue using the human procollagen I antibody to identify human cells. Shown in left hand panel is a low power image demonstrating numerous human cells expressing procollagen I (brown stain) in xenotransplanted mice receiving human CD44^{hi} IPF MPCs. A relative paucity of human cells expressing procollagen I was seen in xenotransplanted mice receiving human CD44^{lo} IPF MPCs (right hand panel). Bottom panels: IHC control using isotype antibody. Scale bars: 500 µm.

Supplementary Figure 4

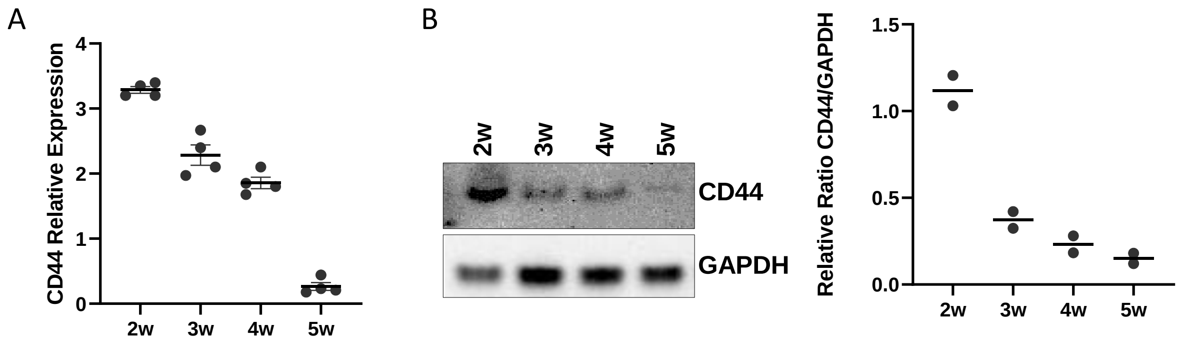

Supplementary Figure 4. CD44 expression declines in CD44^{hi} IPF MPCs as a function of time. A and B. CD44^{hi} IPF MPCs were cultured on tissue culture dishes in DMEM + 10% FBS for up to 5 weeks. CD44 expression was quantified by Q-PCR (**A**) and Western Blot analysis (**B**). Densitometry values are shown in the right hand graph.

Supplementary Figure 5

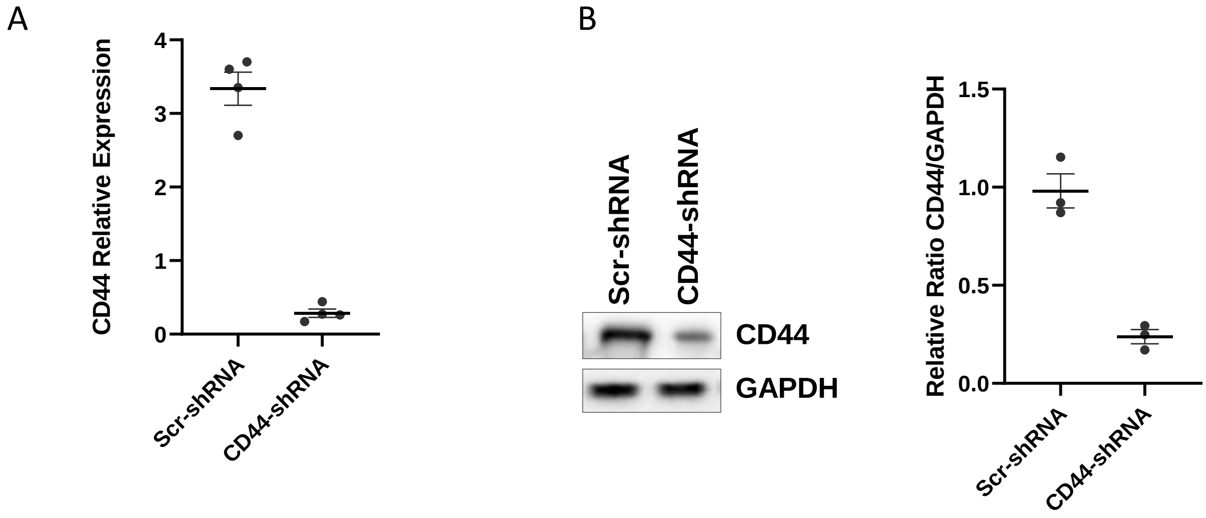

Supplementary Figure 5. CD44 expression levels in CD44^{hi} IPF MPCs transduced with CD44-shRNA or scrambled- shRNA. A and B. CD44^{hi} IPF MPCs transduced with CD44-shRNA or scrambled shRNA (Scr-shRNA). CD44 mRNA and protein expression were quantified by Q-PCR (**A**) or Western Blot analysis (**B**). Densitometry values are shown in the right hand graph.

Supplementary Table 1
CD44 Nuclear Interactome: cell proliferation, adhesion, and migration-related proteins

ACAP3	EFCAB13	MAPKAPK3	SRRM2
ADAR	ESPL1	METTL16	SUGP2
AHNAK	EXOSC10	MGA	SUGT1
ALPK2	FANCC	MTOR	SYNE1
APC	FANCI	MYOF	SYNE2
ARHGAP35	GAR1	NCAPD3	TDRD5
ARID4A	GLE1	NOP14	TESK2
ARID5A	HECTD4	NUMA1	TET1
ASH1L	HIF3A	POLR3B	TNKS1BP1
ASPM	HIST1H1C	PSMC2	TOP3B
ATOH8	HIST1H1E	PTMA	TRANK1
BRCA1	HLTF	RAD54L2	TRRAP
BRCA1	HNRNPM	RBL1	ZEB1
BRCA2	HNRNPUL1	RBM27	ZFAT
BTAF1	HOXA6	RBMX	ZNF140
BUD13	INTS6	RNF20	ZNF227
C2orf42	JMJD1C	RRP36	ZNF235
CAMTA2	KCTD1	RSF1	ZNF318
CBX4	KDM4B	SDAD1	ZNF541
CDK13	KNL1	SETD3	ZNF559
CHD1	KNTC1	SF3B3	ZNF595
CKAP5	KNTC1	SLC8A2	ZNF646
CTDSP1	LCORL	SMARCA4 (BRG1)	ZRANB2
CTR9	LMNA	SPTY2D1	
DDX17	MAMSTR	SRRM2	

Supplementary Table 2

Ensembl_gene_id	Gene_Symbol	Pearson_correlation_coefficient	P-value (uncorrected)
ENSG00000026508	CD44	1	0
ENSG00000112308	C6orf62	0.812825018	1.11E-38
ENSG00000171862	PTEN	0.79730461	2.99E-36
ENSG00000124193	SRSF6	0.796885778	3.45E-36
ENSG00000134294	SLC38A2	0.796300885	4.22E-36
ENSG00000164548	TRA2A	0.788619819	5.56E-35
ENSG00000112081	SRSF3	0.766765897	4.92E-32
ENSG00000143742	SRP9	0.764054364	1.08E-31
ENSG00000189241	TSPYL1	0.756738053	8.68E-31
ENSG00000122566	HNRNPA2B1	0.755204923	1.33E-30
ENSG00000277443	MARCKS	0.750493706	4.84E-30
ENSG00000189403	HMGB1	0.732844344	4.78E-28
ENSG00000116489	CAPZA1	0.728646975	1.35E-27
ENSG00000188612	SUMO2	0.716804666	2.29E-26
ENSG00000165672	PRDX3	0.713839232	4.55E-26
ENSG00000150347	ARID5B	0.712384888	6.34E-26
ENSG00000109133	TMEM33	0.711445383	7.86E-26
ENSG00000108953	YWHAE	0.706617632	2.33E-25
ENSG00000132669	RIN2	0.705532248	2.97E-25
ENSG00000168036	CTNNB1	0.702388742	5.94E-25

Supplementary Table 2 The top 20 genes correlating with CD44 expression
Single-cell RNA expression data from SSEA4^{hi} mesenchymal cells (Mesenchymal Progenitor Cells, MPCs) from control and IPF lung tissue utilizing the Fluidigm C1 platform was obtained from NCBI Bioproject PRJNA641647, and analyzed as previously described (10). Cells originating from IPF origin were extracted (n=159 IPF derived MPCs). The Pearson correlation coefficient and p-values with CD44 expression was calculated for all expressed genes (n=27,061). False discovery rate was calculated using the q-value package (52), and genes with a false discovery rate of <0.01 were considered significant.

Supplementary reference

52. Storey JD, et al. Version 2.22.0. qvalue: Q-value estimation for false discovery rate control; 2015. Accessed March 29, 2021. https://rdrr.io/bioc/qvalue/.
